# Overexpression of the class I homeodomain transcription factor TaHDZipI‐5 increases drought and frost tolerance in transgenic wheat

**DOI:** 10.1111/pbi.12865

**Published:** 2017-12-27

**Authors:** Yunfei Yang, Sukanya Luang, John Harris, Matteo Riboni, Yuan Li, Natalia Bazanova, Maria Hrmova, Stephan Haefele, Nataliya Kovalchuk, Sergiy Lopato

**Affiliations:** ^1^ School of Agriculture, Food and Wine University of Adelaide Glen Osmond SA Australia; ^2^ Present address: Institute of Molecular Biosciences Mahidol University Nakhon‐Pathom Thailand; ^3^ Present address: South Australian Research and Development Institute GPO Box 397 Adelaide SA 5064 Australia; ^4^ Present address: Commonwealth Scientific and Industrial Research Organisation Glen Osmond SA 5064 Australia; ^5^ Present address: Rothamsted Research West Common Harpenden Hertfordshire Al5 2JQ UK

**Keywords:** 3D protein modelling, abiotic stress, activation domain, phenotypic features, protein homo‐ and hetero‐dimerization, stress‐inducible promoters

## Abstract

Characterization of the function of stress‐related genes helps to understand the mechanisms of plant responses to environmental conditions. The findings of this work defined the role of the wheat *TaHDZipI‐5* gene, encoding a stress‐responsive homeodomain–leucine zipper class I (HD‐Zip I) transcription factor, during the development of plant tolerance to frost and drought. Strong induction of *TaHDZipI‐5* expression by low temperatures, and the elevated *TaHDZipI‐5* levels of expression in flowers and early developing grains in the absence of stress, suggests that *TaHDZipI‐5* is involved in the regulation of frost tolerance at flowering. The TaHDZipI‐5 protein behaved as an activator in a yeast transactivation assay, and the TaHDZipI‐5 activation domain was localized to its C‐terminus. The TaHDZipI‐5 protein homo‐ and hetero‐dimerizes with related TaHDZipI‐3, and differences between DNA interactions in both dimers were specified at 3D molecular levels. The constitutive overexpression of *TaHDZipI‐5* in bread wheat significantly enhanced frost and drought tolerance of transgenic wheat lines with the appearance of undesired phenotypic features, which included a reduced plant size and biomass, delayed flowering and a grain yield decrease. An attempt to improve the phenotype of transgenic wheat by the application of stress‐inducible promoters with contrasting properties did not lead to the elimination of undesired phenotype, apparently due to strict spatial requirements for *TaHDZipI‐5* overexpression.

## Introduction

Drought and frost are significant limitations to plant growth and development and substantially decrease crop yields globally, including in Australia. Depending on seasonal conditions, a sudden frost at flowering can be a major cause of wheat and barley grain yield losses. Overnight frost events during flowering will damage the sensitive reproductive tissues, often resulting in a near‐total loss of grain. If crops are sown late with the aim to avoid productivity losses due to frost, severe yield losses may occur in hot and dry periods at the end of the growing season. In addition, late planting often leads to reduced grain size, yield and quality. The cost to Australian wheat and barley industries caused by frost is estimated to be around AUS$ 360 million in direct and indirect losses annually (GRDC National Frost Initiative, goo.gl/hKWf34). Thus, identification, characterization and application of candidate genes for the molecular breeding of crop acclimation to both frost and drought are of the utmost importance.

Environmental stresses such as frost or drought trigger specific signal transduction pathways, which activate the expression of stress‐responsive genes (Braam *et al*., 1997; Bray, 1997; Hwang *et al*., 2002; Tena *et al*., 2001; Zhu, 2016). Gene expression starts from the modulation of transcription by stress‐related transcription factors (TFs), which regulate a number of physiological processes under stress, including cuticular wax biosynthesis (Aharoni *et al*., 2004; Bi *et al*., 2016, 2017; Borisjuk *et al*., 2014; Seo *et al*., 2011), stomatal closure (Ren *et al*., 2010; Tan *et al*., 2017), reactive oxygen species (ROS) detoxification (Jiang and Deyholos, 2009) and structural alterations in plasma membranes (Pearce, 1999). Manipulation using genes encoding stress‐related TFs offers the possibility to regulate large groups of genes involved in the same physiological processes, and therefore, this intervention draws the attention of plant biotechnologists (Agarwal *et al*., 2017; Gahlaut *et al*., 2016; Hrmova and Lopato, 2014).

An attractive target for this approach is the family of homeodomain–leucine zipper (HD‐Zip) TFs, which contains proteins regulating plant development after plants are exposed to environmental stimuli and stresses (Brandt *et al*., 2014; Harris *et al*., 2011; Perotti *et al*., 2017). All HD‐Zip TFs possess a highly conserved homeodomain (HD) and leucine zipper (Zip or LZ) motifs (Ariel *et al*., 2007; Harris *et al*., 2016; Mattsson *et al*., 1992; Ruberti *et al*., 1991; Schena and Davis, 1992, 1994). An HD is a folded helix‐turn‐helix motif, which contains 60 amino acid residues (Gehring *et al*., 1990; Laughon and Scott, 1984; Otting *et al*., 1990) and functions during the recognition of specific DNA sequences (Gehring *et al*., 1990; Shepherd *et al*., 1984). LZ, adjacent to HD, participates in dimerization of HD‐Zip TFs by forming a coiled coil structure (Harris *et al*., 2016; Ruberti *et al*., 1991; Szilák *et al*., 1997). Dimerization could affect the affinity of HD‐Zip proteins to specific DNA binding sites and hence potentially regulate the strength of activation of target genes (Chew *et al*., 2013; Harris *et al*., 2016; Palena and Gonzalez, 1999; Szilák *et al*., 1997).

The HD‐Zip family of proteins has been classified into four subfamilies, designated HD‐Zip classes I to IV, based on unique features in the domain structure and specificity of *cis*‐element binding (Ariel *et al*., 2007). The members of HD‐Zip class I differ from the other family members by the absence of common domains and/or motifs besides the HD and Zip domains (Ariel *et al*., 2007; Chan *et al*., 1998; Mukherjee and Bürglin, 2006; Ponting and Aravind, 1999; Schrick *et al*., 2004). The members of the HD‐Zip I family recognize a specific 9‐bp pseudo‐palindromic binding site: CAATNATTG (Meijer *et al*., 1997; Sessa *et al*., 1993). No obvious requirements for the central nucleotide of the *cis*‐element have been observed for wheat HD‐Zip I TFs (Harris *et al*., 2016; Kovalchuk *et al*., 2016). However, it is not clear how homo‐ or hetero‐dimerization of HD‐Zip I TFs influences DNA binding and the activation of target genes (Chew *et al*., 2013; Harris *et al*., 2016; Hrmova and Lopato, 2014).

Homeodomain–leucine zipper class I TFs were isolated from a variety of species such as *Arabidopsis thaliana* (Ariel *et al*., 2007; Schena and Davis, 1992), resurrection plant *Craterostigma plantagineum* (Deng *et al*., 2002; Frank *et al*., 1998), sunflower (Cabello and Chan, 2012; Cabello *et al*., 2012), rice (Agalou *et al*., 2008), maize (Zhao *et al*., 2011) and wheat (Harris *et al*., 2016; Lopato *et al*., 2006). Some of these TFs have been reported to respond to various abiotic stresses on transcriptional and/or post‐translational levels (Bhattacharjee *et al*., 2016; Harris *et al*., 2016; Kovalchuk *et al*., 2016; Olsson *et al*., 2004; Wu *et al*., 2016; Zhao *et al*., 2014). For instance, transcription of *Athb7* and *Athb12* from *Arabidopsis* was induced by elevated levels of abscisic acid (ABA) and by water deficiency (Olsson *et al*., 2004; Söderman *et al*., 1996). Transcription of *Hahb1* from sunflower was responsive to low temperatures (Cabello *et al*., 2012), while *Hahb4* was activated by desiccation (Dezar *et al*., 2005a,b). Transcription of wheat *TaHDZipI‐2* was not influenced by ABA and was partially suppressed by low temperatures; however, the transactivation activity of the TaHDZipI‐2 protein was strongly increased by the addition of exogenous ABA (Kovalchuk *et al*., 2016). In contrast, *TaHDZipI‐4* and *TaHDZipI‐5* were activated by ABA on both transcriptional and post‐translational levels (Harris *et al*., 2016).

The effect of overexpression of HD‐Zip TFs on the ability of transgenic plants to survive severe stress conditions has also been demonstrated (Bhattacharjee *et al*., 2016; Cabello *et al*., 2012; Cabello and Chan, 2012; Kovalchuk *et al*., 2016; Wu *et al*., 2016; Zhang *et al*., 2012). For instance, overexpression of the *Oshox22* and *Oshox24* genes (HD‐Zip I γ‐clade) in transgenic rice and *Arabidopsis* led to an increased ABA content and increased sensitivity to drought and high salinity (Bhattacharjee *et al*., 2016, 2017; Zhang *et al*., 2012). In contrast, overexpression of the similar *ZmHDZ4* gene (HD‐Zip I γ‐clade) from maize in transgenic rice enhanced plant tolerance to drought, despite an increased sensitivity to ABA. *ZmHDZ4*‐expressing transgenic plants had a lower relative electrolyte leakage, lower malondialdehyde levels and increased proline contents under drought compared to wild‐type (WT) plants (Wu *et al*., 2016). All of these changes could potentially contribute to enhanced drought tolerance.

Improvement of cold/frost tolerance was demonstrated only for the representatives of the HD‐Zip I α‐clade. Constitutive overexpression of *AtHB13* from *Arabidopsis* and *HaHB1* from sunflower had little influence on the growth and yield of transgenic *Arabidopsis*, but stabilized cell membrane integrity under cold, drought and high salinity conditions and increased plant stress tolerance (Cabello and Chan, 2012; Cabello *et al*., 2012). Frost tolerance enhancement of transgenic barley seedlings was achieved by constitutive overexpression of *TaHDZipI‐2*, the wheat orthologue of *AtHB13*. However, it was accompanied by negative changes in the phenotype of transgenic plants and a significant yield loss compared to those of control plants (Kovalchuk *et al*., 2016).

In our previous projects, five genes encoding the members of HD‐Zip subfamily I TFs, designated *TaHDZipI‐1* to *TaHDZipI‐5*, were isolated from wheat and partially characterized (Harris *et al*., 2016; Kovalchuk *et al*., 2016; Lopato *et al*., 2006). *TaHDZipI‐1* (*ζ*‐clade) expression was detected in seedlings and mature vegetative tissues, while *TaHDZipI‐2* (α‐clade) was predominantly expressed in shoots of seedlings and during early grain development, with no expression detected in mature tissues (Lopato *et al*., 2006). *TaHDZipI‐2* was demonstrated to function as a regulator of plant growth, flowering time and frost tolerance (Kovalchuk *et al*., 2016). Overexpression of *TaHDZipI‐2* in transgenic barley directly or indirectly regulated a number of genes responsible for barley adaptation to cold, vernalization, flowering time and shape of spikes (Kovalchuk *et al*., 2016). The *TaHDZipI‐3* gene (γ‐clade) was initially identified as a close homologue of *AtHB7* and *AtHB12* from *Arabidopsis*, and its induction of transcription by drought was demonstrated by Harris *et al*. (2016). In contrast to the homologous genes from *Arabidopsis*,* TaHDZipI‐3* was not activated by cold and was not able to function as an activator in yeast or in wheat cells. Therefore, the full‐length coding region of this protein provided ideal bait for a yeast 2‐hybrid (Y2H) screen. The screen identified two interacting partners, which were identified to be the monocot‐specific members of the γ‐clade, designated *TaHDZipI‐4* and *TaHDZipI‐5*. In contrast to *TaHDZipI‐3*, transcription of both monocot‐specific genes was ABA‐dependent and was strongly up‐regulated by both cold and drought (Harris *et al*., 2016).

This study is directed to identify regulatory genes that could be used for the improvement of frost and drought tolerance in economically important plants, such as wheat and barley. *TaHDZipI‐5* was selected for further characterization because it was more strongly induced by cold and drought than the two other genes from the wheat HD‐Zip I γ‐clade (Harris *et al*., 2016). In this work, we studied the expression levels of *TaHDZipI‐5* in a variety of wheat tissues, analysed the TaHDZipI‐5 transactivation properties and revealed the determinants of homo‐ and hetero‐dimerized TaHDZipI‐5 and TaHDZipI‐3 in complex with a defined *cis‐*element at the 3D molecular level. *TaHDZipI‐5* was initially constitutively overexpressed in transgenic wheat, and comparative evaluations of transgenic and WT plants for growth characteristics and yield components, and tolerance to extreme stress conditions were performed. Overexpression of *TaHDZipI‐5* significantly improved plant tolerance to both stresses; however, it negatively influenced plant growth and grain yields. Stress‐inducible expression of *TaHDZipI‐5* was applied in an attempt to reduce the negative influence of the transgene on plant development, the onset of flowering and yield.

## Results

### A reconstruction of the phylogenetic relationship of HD‐Zip I γ‐clade proteins

**Figure 1:**
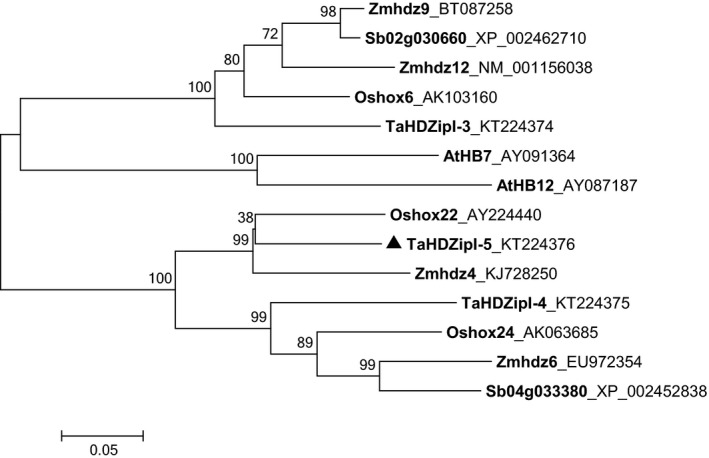
A rectangular phylogenetic tree displaying the evolutionary relationships of HD‐Zip I γ‐clade TFs from *Arabidopsis* and selected monocots. Abbreviations of species: At, *Arabidopsis thaliana*; Os, *Oryza sativa*; Sb, *Sorghum bicolor*; Ta, *Triticum aestivum*; Zm, *Zea mays*.

A phylogenetic tree was constructed using sequences of HD‐Zip I γ‐clade proteins from the dicot model plant *Arabidopsis* and from several monocots including sorghum, rice, maize and wheat. Protein sequences were either derived from a previous study (Henriksson *et al*., 2005) or were taken from NCBI databases and compared with the translated sequence of *TaHDZipI‐5* (Table S2). The phylogenetic tree (Figure 1) shows that TaHDZipI‐5 shares a closer evolutionary relationship with Oshox22 from rice (69% sequence identity) and Zmhdz4 from maize (63% sequence identity), than with other entries in the tree.

### Endogenous *TaHDZipI‐5* expression in a variety of unstressed bread wheat tissues

**Figure 2:**
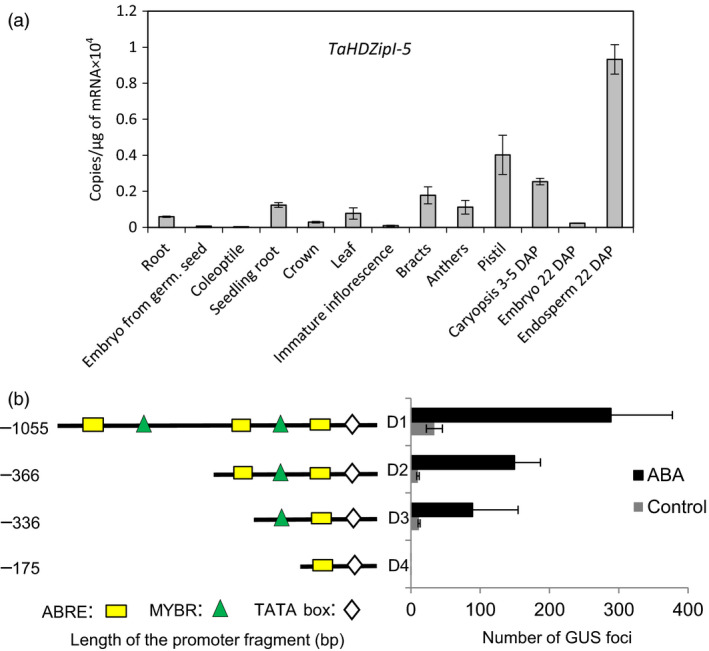
Characterization of the *TaHDZipI‐5* gene. (a) Transcript numbers of the *TaHDZipI‐5* gene in wheat tissues were estimated by Q‐PCR. (b) Mapping of *cis*‐elements responsible for the abscisic acid (ABA)‐dependent activation of the *TdHDZipI‐5A* promoter, using a transient expression assay in wheat cell culture. Depicted is a schematic representation of ABA‐responsive element (ABRE) and MYB responsive (MYBR) *cis*‐elements in four promoter deletions (D1–D4) of the *TdHDZipI‐5A* promoter, and the graph shows activation of GUS expression by the deletions detected in a transient expression assay, in the presence (black bars) or absence (control; grey bars) of 0.5 mm ABA in the culture medium. Error bars were calculated from three technical replicates.

Expression of the endogenous *TaHDZipI‐5* gene was analysed in a variety of tissues of unstressed wheat plants. *TaHDZipI‐5* had the highest expression level in endosperm (Figure 2a). Additionally, high expression levels were found in roots and reproductive plant tissues sampled around fertilization. The lowest expression levels of *TaHDZipI‐5* were detected in coleoptiles; these were about 30‐fold lower than those in the endosperm.

### Functional *cis*‐elements responsible for ABA‐dependent *TdHDZipI‐5* promoter activation

Promoter sequences of two homeologous genes, *TdHDZipI‐5A* and *TdHDZipI‐5B*, were isolated from a durum wheat BAC library (Cenci et al., 2003), because the respective bread wheat sequences were not yet available at the time when this work commenced. The comparison of durum wheat promoter sequences (*TdHDZipI‐5A* and *TdHDZipI‐5B*) with corresponding sequences from bread wheat, identified in the Whole Genome Reference Assembly Pseudomolecules v1.0 databases of the International Wheat Genome Sequencing Consortium (IWGSC), revealed more than 99% sequence identity in a region containing functional *cis*‐elements (Figure S3). Sequences of the promoters (each approximately 1300 bp long) were aligned using LALIGN (Huang and Miller, 1991) to find the best local alignments (Figure S4). Several ABRE and MYB responsive elements were predicted in conserved positions in both *TdHDZipI‐5* promoter regions (PLACE software; Higo *et al*., 1999). *TdHDZipI‐5A* promoter deletions were generated based on putative *cis‐*acting elements at −1055, −366, −336 and −175 bp positions, and these were named D1, D2, D3 and D4 (Figures 2b and S4). To define the functional *cis*‐elements, 0.5 mm ABA was used to induce the activation of the *GUS* reporter gene by four promoter deletions in a transient expression assay performed in cultured wheat cells. Transformation with D1, D2 and D3 led to step‐by‐step decreasing numbers of GUS foci, while D4 could not activate *GUS* gene expression (Figure 2b). Therefore, the putative *cis‐*element responsible for the ABA‐dependent activation of *TdHDZipI‐5A* is the MYB responsive (MYBR) element GGATA, which is located in the 161‐bp region between D3 (−336 np) and D4 (−175 np), upstream of the transcription initiation site (Figure 2b). Two upstream ABA‐responsive elements (ABREs) and/or one MYBR element enhanced the ABA‐inducible promoter activation.

### Identification of the TaHDZipI‐5 activation domain using an in‐yeast activation assay

**Figure 3:**
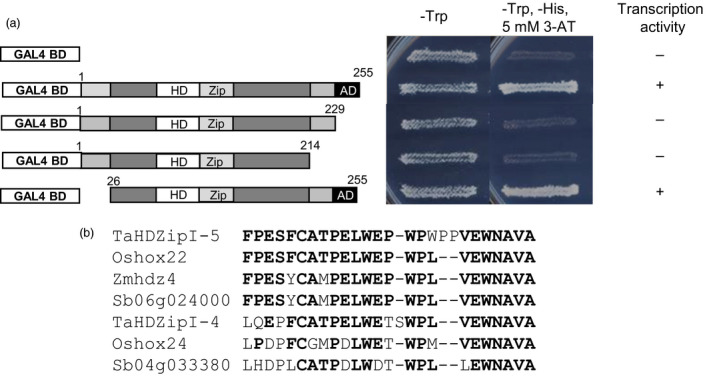
Identification of the TaHDZipI‐5 activation domain (AD) using an in‐yeast activation assay. (a) Transcription activity determined through the in‐yeast activation assay, where homeodomain (HD), Zip and AD designate putative homeodomain, leucine zipper and AD, respectively. Amino acid residues at the beginning and end of each truncated protein are indicated with numbers. (b) Conserved sequences of the identified AD in TaHDZipI‐5 and close homologues from other monocots. Ta, *Triticum aestivum*; Zm, *Zea mays*; Os, *Oryza sativa*; Sb, *Sorghum bicolor*. Amino acid residues, which are the same in more than the half of the investigated sequences, are in bold.

Results of the in‐yeast activation assay showed that the yeast strains carrying pGBKT7‐TaHDZipI‐5 grew well on SD/‐Trp medium (confirming transformation) and the SD/‐Trp/‐His medium containing 5 mm 3‐AT (confirming the yeast *HIS3* reporter gene activation by a plant activation domain‐AD) (Figure 3a). To locate the position of the TaHDZipI‐5 AD, different truncated variants of the TaHDZipI‐5 protein were tested with the in‐yeast activation assay. The empty pGBKT7 vector was transformed into yeast and used as a negative control. After 4 days of cultivation, the yeast carrying the 26–255 residue fragment grew well on SD/‐Trp/‐His medium, while the yeast carrying the 1–229 aa and 1–214 residue fragments were not able to grow on the selective medium, suggesting that the putative AD localizes to the C‐terminal part of the protein (amino acid residues 229–255) (Figure 3a). The identified AD region is represented by a C‐terminal sequence that is conserved in TaHDZipI‐5 homologues from other monocot plants (Figure 3b).

### Based on molecular model predictions of TaHDZipI‐5, homo‐dimerization and hetero‐dimerization influence the DNA binding specificity

Harris *et al*. (2016) have recently shown that the expression levels of *TaHDZipI‐4* and *TaHDZipI‐5* increased under cyclic drought conditions, while those of *TaHDZipI‐3* remained low. These authors proposed a model explaining how TaHDZipI‐3 binds to DNA *cis*‐elements in a homo‐dimeric form and that a hetero‐dimeric form of TaHDZipI‐4 and TaHDZipI‐5 (also members of the class I γ‐clade HDs) would initiate a stress response. In the current work, we compared 3D models of homo‐dimeric TaHDZipI‐3 and TaHDZipI‐5, and of hetero‐dimeric TaHDZipI‐3/TaHDZipI‐5, in complex with a HDZ1 *cis*‐element to seek whether DNA interactions differed between homo‐ and hetero‐dimeric structural models.

**Figure 4:**
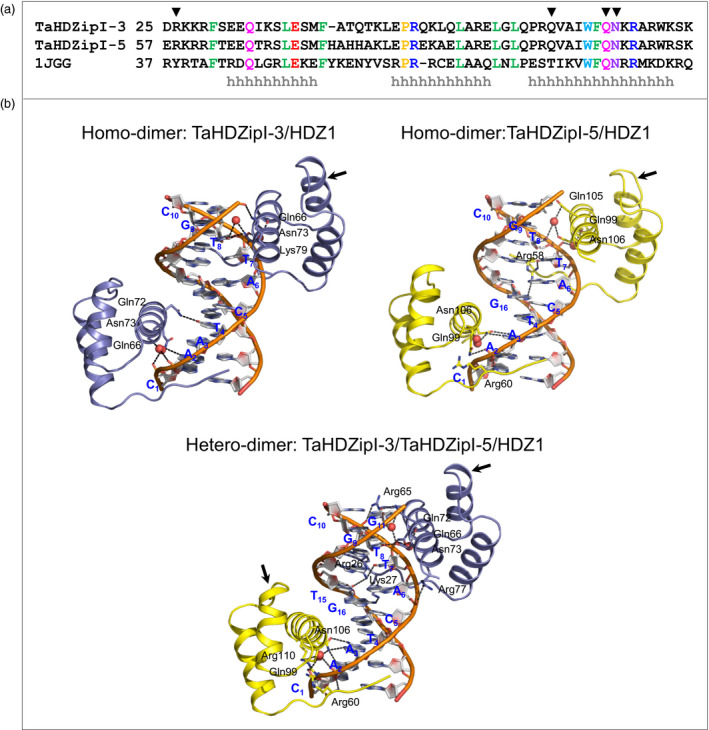
Molecular features of homeodomains (HDs) of TaHDZipI‐3 and TaHDZipI‐5 in homo‐ and hetero‐dimeric forms in complex with the HDZ1 *cis*‐element. (a) A sequence alignment of TaHDZipI‐3 and TaHDZipI‐5 HDs and of even‐skipped HD from *Drosophila melanogaster* (PDB: 1JGG). Identical amino acid residues are coloured based on their properties. α‐Helical secondary structural elements are indicated with ‘h’ below the sequences. HD residues that interact with DNA *cis*‐elements are indicated by inverted triangles (▼). (b) Ribbon representations of homo‐dimeric TaHDZipI‐3 and TaHDZipI‐5, and hetero‐dimeric TaHDZipI‐3/TaHDZipI‐5 models in complex with the HDZ1 *cis*‐element; blue descriptions and atomic colour representations are used for HDZ1. The ribbons of TaHDZipI‐3 and TaHDZipI‐5 are coloured in blue and yellow, respectively. DNA‐interacting residues are shown in sticks, and DNA sugar‐phosphate backbones are coloured in cpk‐orange. Water molecules are shown as red spheres. Interactions (less than 3.5 Å; Table S3) between residues and HDZ1 are shown in black dashed lines. Arrows point to differences in folding of α‐helices between TaHDZipI‐3 and TaHDZipI‐5.

Structural bioinformatic comparisons of wheat TaHDZipI‐3 and TaHDZipI‐5 proteins showed the presence of HD domains with well‐defined boundaries. An alignment of the template (PDB: 1JGG) used for structural modelling and TaHDZipI‐3 and TaHDZipI‐5 indicated that there was not a high level of sequence identity between the investigated proteins (Figure 4, top panel). HDs of TaHDZipI‐3 shared respective 26% identity and 53% similarity to the template (PDB: 1JGG), while TaHDZipI‐3 and TaHDZipI‐5 between themselves shared 31% identity and 52% similarity. The positions of 15 identical residues between the template and target sequences of HDs (Figure 4a) indicated which residues might participate in DNA binding. Secondary structure element predictions in TaHDZipI‐3 and TaHDZipI‐5 indicated the presence of three α‐helices (marked as ‘h’ in Figure 4, top panel) that carried most of these identical residues.

Homo‐dimeric (TaHDZipI‐3/TaHDZipI‐3 or TaHDZipI‐5/TaHDZipI‐5) and hetero‐dimeric (TaHDZipI‐3/TaHDZipI‐5) HD models in complex with HDZ1 (5′‐CAATCATTGC‐3′/5′‐GCAATGATTG‐3′; interacting nucleobases are underlined) (Sali and Blundell, 1993) were constructed to understand the differences in binding of DNA. The structural models of HDs of TaHDZipI‐5 showed the presence of three α‐helices, interconnected with loops similarly to TaHDZipI‐3 (Figure 4, bottom panel); however, minor structural differences were observed between TaHDZipI‐3 and TaHDZipI‐5 (*cf*. arrows in Figure 4, bottom panel). Data from structural modelling indicated that differences in DNA binding to *cis*‐elements resulted from differences in the presence of charged and polar residues at the N‐terminus and at the third α‐helix of each HD monomer that contacted DNA at a major groove (Figure 4, bottom panel; Table S3).

More specifically, our molecular analysis showed that Arg58 in TaHDZipI‐5 at the N‐terminus of the first HD monomer bound directly to two nucleobases T7 and G16, while Gln105 and Asn106 contacted the T8 nucleobase indirectly through a water molecule (Figure 4 bottom panel). In addition, Asn106 in TaHDZipI‐5, which corresponds to Asn73 in TaHDZipI‐3, bound to the nucleobase A3. Further, the analyses of HDZ1‐binding modes in TaHDZipI‐3/TaHDZipI‐5 showed that two positively charged residues of the TaHDZipI‐3 monomer at the N‐terminus bound to the nucleobases T7 and T15 *via* Arg26 and to the nucleobase G16 through Lys27. Additionally, Asn73 at the major groove α‐helix bound to the nucleobase T8, while Gln72 contacted T8 *via* a water molecule and could also form a hydrogen bond to the nucleobase G11. On the other hand, the interactions of the TaHDZipI‐5 HD monomer with HDZ1 were similar to those of the second HD monomer in the homo‐dimeric form.

A detailed analysis of hydrogen bond patterns in homo‐dimeric (TaHDZipI‐5/TaHDZipI‐5) and hetero‐dimeric (TaHDZipI‐3/TaHDZipI‐5) DNA complexes (Table S3) showed that three nucleobases (A2, T4 and T8) were bound to Asn73 (participating through both monomers) and Gln72 (Harris *et al*., 2016). Besides, four nucleobases (A3, T7, T8 and G16) were bound to Arg58, Gln105 and Asn106 (participating through both monomers) in homo‐dimeric TaHDZipI‐5. The analysis of interactions in hetero‐dimeric TaHDZipI‐3/TaHDZipI‐5 showed the participation of seven nucleobases (A2, A3, T7, T8, G11, T15 and G16) that were contacted through Arg26, Lys27, Gln72, Asn73 of TaHDZipI‐3 and through Asn106 of TaHDZipI‐5; Arg26 and Gln72 residues formed bidentate interactions with DNA. Free energies of homo‐dimeric TaHDZipI‐5 (290 kcal/mol) and hetero‐dimeric TaHDZipI‐3/TaHDZipI‐5 (244 kcal/mol), calculated through FoldX (Schymkowitz *et al*., 2005), indicated that hetero‐dimeric TaHDZipI‐3/TaHDZipI‐5 was more stable than its TaHDZipI‐5 homo‐dimeric form.

### Evaluation of transgenic wheat plants constitutively expressing *TaHDZipI‐5*

Initially, transgenic wheat plants (cv. Gladius) were generated using a construct where expression of the transgene *TaHDZipI‐5* was driven by a constitutive maize polyubiquitin promoter. Three independent transgenic T_1_ lines, L1, L2 and L4 containing a single copy of the transgene (Figure S5), were used for characterization of plant phenotypes and yield components under well‐watered conditions. Two of three transgenic lines showed a very similar phenotype to that of WT plants (Figure S6). However, L1 plants were significantly shorter than WT plants; they had a significantly lower seed number per spike and a lower grain yield than WT plants (Figure S6). All transgenic lines showed delay in flowering time compared to that of WT plants (Figure S6).

**Figure 5:**
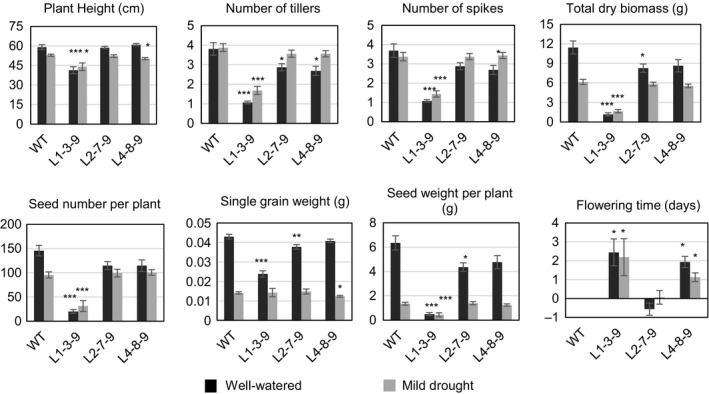
Growth characteristics and yield components of control wild‐type (WT) (*Triticum aestivum* cv. Gladius) and T_3_ transgenic wheat transformed with pUbi‐TaHDZipI‐5 under well‐watered (black boxes) and under drought conditions (grey boxes). Flowering time of transgenic plants was compared to the average flowering time of 16 control WT plants, which is represented as day 0. Differences between transgenic lines and WT plants in each of the well‐watered and drought conditions were tested using unpaired Student's *t*‐tests (*P < 0.05, **P < 0.01, ***P < 0.001).

The T_3_ progeny of transgenic and WT plants were grown in two large containers with different watering regimes (Figure S1; Table S4). Using pilot experiments described in the Materials and Methods, all three starting T_2_ lines, L1‐3‐9, L2‐7‐9 and L4‐8‐9 (pUbi‐TaHDZipI‐5), were identified to be homozygous for the transgene. The phenotypic data of transgenic wheat lines were compared to those of WT plants (Figure 5). The data obtained from the well‐watered bin correlated well with those obtained for T_1_ transgenic plants grown in pots. The progeny of L1‐3‐9 had reduced plant height, less dry biomass, fewer tillers, spikes and seeds, and about 90% lower grain yield (seed weight per plant) than WT plants. The other two lines showed up to a 25%–30% decrease in all parameters compared with those of WT plants, except for flowering time where differences did not exceed 2 days (Figure 5). In contrast to the data obtained under well‐watered conditions, the differences in growth characteristics and yield parameters between progenies of these two lines (L2‐7‐9 and L4‐8‐9) and WT plants were small under drought (Figure 5).

### Drought tolerance (survival) of transgenic wheat seedlings was significantly higher than that of WT plants

**Figure 6:**
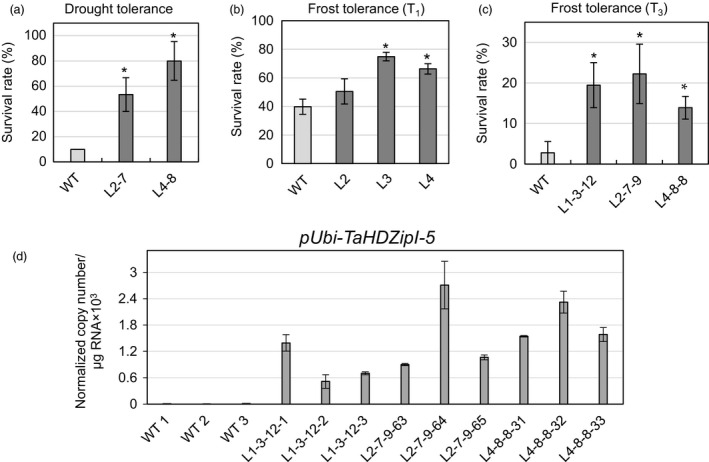
Comparison of drought and frost tolerance of wild‐type (WT) (*Triticum aestivum* cv. Gladius) and transgenic wheat transformed with pUbi‐TaHDZipI‐5. (a) Drought tolerance of two independent transgenic lines (T_2_ progeny), sizes of which were similar to those of the control WT plants’, is shown as the survival rate of plants recovered after the terminal drought stress, followed by rewatering. (b, c) Survival rates of plants recovered after seedling‐stage frost: (b) T_1_ progeny and (c) T_3_ progeny of transgenic plants. Error bars represent ± SD of three independent experiments. Values represent means ± SE (*n* varies for each column and is shown in each case directly on the graphs). Significant differences between transgenic lines and WT plants were tested using an unpaired Student's *t*‐test (*P < 0.05). (d) The expression levels of the *TaHDZipI‐5* transgene in leaves of T_3_ plants in unstressed control WT and transgenic plants, sampled prior to frost treatment. Error bars represent ± SD of three technical replicates.

Three‐week‐old transgenic wheat seedlings (T_2_ progenies of sublines L2‐7 and L4‐8 transformed with pUbi‐TaHDZipI‐5, and WT plants, ten plants for each class) were used in three independent drought tolerance experiments. We only examined lines with minimal phenotypic differences compared to those of WT plants. Two control plants and two transgenic plants from each line were planted in the same pot with the aim to minimize influence of differences in seedling sizes and respective differences in water consumption on water availability in the soil. Only 10% of WT plants survived the applied drought conditions and recovered after rewatering (Figure 6a). In contrast, both tested transgenic lines showed a significantly stronger ability to recover than WT plants, with over half of all tested plants surviving (Figure 6a).

### Transgenic wheat seedlings tolerate frost better than WT plants

Three‐week‐old T_1_ (Figure 6b) and T_3_ (Figure 6c) generations of transgenic seedlings were used in frost tolerance tests. Twelve plants of each transgenic line and twelve WT plants were treated in a semi‐automated cold cabinet, using an updated program (Figure S2) based on the earlier established protocol for barley (Kovalchuk *et al*., 2013). This included 6.5‐h exposure to −7 °C for the T_1_ generation and the same exposure time to −8 °C for T_3_ plants; both generations were tested in three independent experiments. All tested transgenic lines showed a higher survival rate than those of WT plants in both experiments (Figure 6b,c). Levels of transgene expression were determined by Q‐PCR using RNA isolated from unstressed wheat leaves collected from transgenic and control plants before frost tolerance tests were conducted (Figure 6d).

### Evaluation of transgenic wheat plants with stress‐inducible expression of *TaHDZipI‐5*

**Figure 7:**
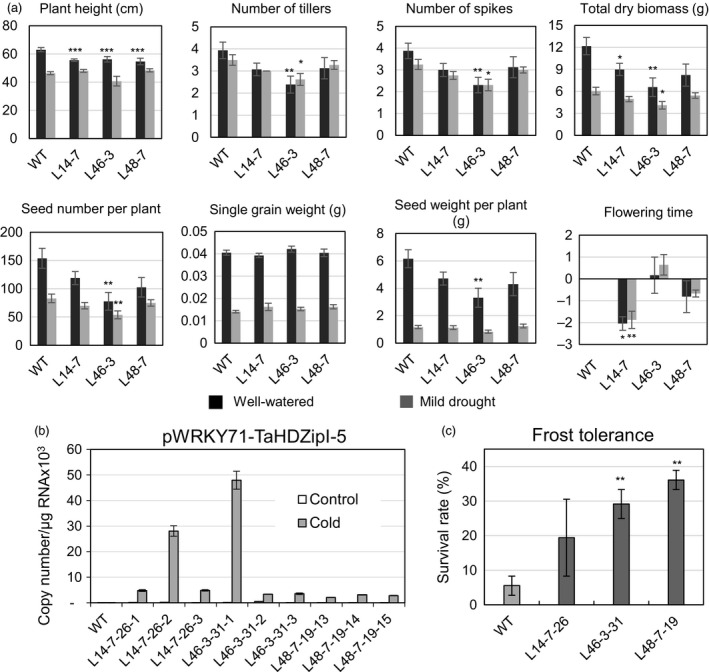
Characteristics of transgenic wheat transformed with pWRKY71‐TaHDZipI‐5. (a) Comparisons of plant growth and yield characteristics of wild‐type (WT) and transgenic T_2_ plants under well‐watered conditions (black boxes) and mild drought (grey boxes). (b) Transgene expression levels in control WT and transgenic T_4_ plants at 23 °C (control) and 4 °C (cold). (c) Frost tolerances of WT and transgenic wheat transformed with pWRKY71‐TaHDZipI‐5 are shown as the survival rate of plants recovered after the terminal frost treatment. Error bars represent ± SD for three independent experiments. Differences between transgenic lines and WT plants were tested in the unpaired Student's *t*‐test (*P < 0.05, **P < 0.01, *** for *P* < 0.001).

**Figure 8:**
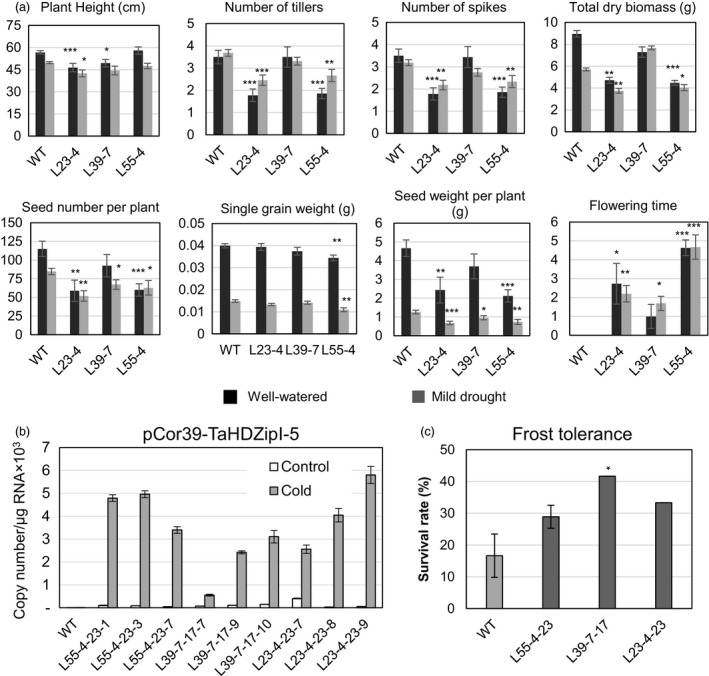
Characteristics of transgenic wheat transformed with pCor39‐TaHDZipI‐5. (a) Comparison of plant growth and yield characteristics of wild‐type (WT) and transgenic T_2_ plants, under well‐watered conditions (black boxes) and mild drought (grey boxes). (b) Transgene expression levels in WT and transgenic T_4_ plants at 23 °C (control) and 4 °C (cold). (c) Frost tolerance of WT and transgenic wheat transformed with pCor39‐TaHDZipI‐5 is shown as the survival rate of plants recovered after the terminal frost treatment. Error bars represent ± SD for three independent experiments. Differences between transgenic lines and WT plants were tested in the unpaired Student's *t*‐test (*P < 0.05, **P < 0.01, *** for *P* < 0.001).

With the aim to avoid or minimize the influence of *TaHDZipI‐5* overexpression on the developmental phenotype of transgenic wheat, we replaced the *ZmUbiquitin* constitutive promoter with one of the stress‐inducible promoters from *OsWRKY71* and *TdCor39* genes. These stress‐inducible promoters were previously employed to avoid the negative influences of *TaDREB3* gene expression on phenotype and yield in transgenic barley (Kovalchuk *et al*., 2013). Generation of transgenic plants, selection of homozygous lines, assessment of yield components and evaluation of frost tolerance were performed similarly as for transgenic wheat plants transformed with the pUbi‐TaHDZipI‐5 construct (Figures 7 and 8). Surprisingly, cold‐inducible expression of the transgene led to a reduced difference between flowering time of transgenic and WT plants only for the plants with the *pWRKY71* promoter, whereas both stress‐inducible promoters, *pWRKY71* and *pCor39*, stabilized the single grain weight in transgenics (Figures 7a and 8a). Although both promoters showed the low levels of basal promoter activity in unstressed transgenic wheat plants (Figures 7b and 8b), many of the negative phenotype and yield characteristics failed to improve, compared to transgenic plants with constitutive transgene expression. Nevertheless, both transgenics demonstrated the substantial enhancement of frost tolerance during the vegetative developmental stage (Figures 7c and 8c).

## Discussion

The *TaHDZipI‐*5 cDNA was isolated in a Y2H screen using *TaHDZipI‐3* as bait, from a cDNA library prepared from flag leaves and spikes of *Triticum aestivum* L. genotype RAC875, subjected to drought and heat stresses. Subsequently, the gene and gene product were characterized at the molecular level (Harris *et al*., 2016). *TaHDZipI‐5* expression was induced by drought, frost and ABA treatment. Based on a close evolutionary relationship with the *Arabidopsis* γ‐clade of the HD‐Zip I subfamily and conserved intron/exon structure, the *TaHDZipI‐5* gene was identified as a monocot‐specific member of the γ‐clade (Harris *et al*., 2016). The closest homologues of *TaHDZipI‐5* from rice and maize, *Oshox22* and *Zmhdz4*, participate in ABA‐mediated drought response, and expression of these genes is up‐regulated by water deficiency (Wu *et al*., 2016; Zhang *et al*., 2012).

The analysis of transgenic rice and *Arabidopsis* plants revealed that constitutive overexpression of *Oshox22* and another monocot‐specific γ‐clade member from rice, *Oshox24*, leads to negative regulation of response to drought/dehydration and, hence, to increased sensitivity of transgenic plants to drought (Bhattacharjee *et al*., 2016, 2017; Zhang *et al*., 2012). In contrast, overexpression of the very similar gene from maize, *Zmhdz4,* positively regulated plant responses to stress in transgenic *Arabidopsis* and conferred tolerance to drought in transgenic rice (Wu *et al*., 2016).

Regulation of HD‐Zip I gene expression by low temperatures was demonstrated for *Arabidopsis* (Cabello *et al*., 2012), tomato (Zhang *et al*., 2014), paper mulberry (Peng *et al*., 2015), sunflower (Cabello *et al*., 2012), rice (Zhang *et al*., 2012) and wheat (Harris *et al*., 2016). The overexpression of α‐clade HD‐Zip class I TFs conferred cold/frost tolerance to transgenic *Arabidopsis* and barley (Cabello *et al*., 2012; Kovalchuk *et al*., 2016); however, the influence on the overexpression of monocot‐specific γ‐clade members on cold or frost tolerance of transgenic plants remains to be determined.

The molecular characterization of the *TaHDZipI‐5* gene and its protein product by Harris *et al*. (2016) and in this work demonstrated its role in wheat tolerance to drought and frost. These studies were conducted through overexpression of the *TaHDZipI‐5* gene in transgenic wheat and the evaluation of transgenic plants. We compared phenotypes and yield components of transgenic and control WT wheat plants under optimal growth conditions and under a slowly increasing drought. The ultimate aims of this work were to optimize transgene performance, decrease negative influences of the transgene on plant development using stress‐inducible promoters and select the optimal homozygous lines for field trials in Australian agricultural regions that are prone to seasonal frosts and long periods of drought (annual and monthly potential frost days; http://www.bom.gov.au/jsp/ncc/climate_averages/frost/index.jsp).

The analysis of *TaHDZipI‐5* expression in a variety of wheat tissues revealed that the level of this gene was elevated in flowers, developing grain and particularly in the endosperm, a plant tissue that contains increased ABA. In contrast, the number of *TaHDZipI‐5* transcripts in vegetative tissues was low (Figure 2a). Expression of *TaHDZipI‐5* in flowers shortly before fertilization, and during the early stages of grain development, and the strong induction of *TaHDZipI‐5* expression by low temperatures possibly suggest the involvement of this gene in the protection of wheat tissues that are most vulnerable to night frosts.

To understand the function of the *TaHDZipI‐5* gene, we isolated gene promoters and revealed DNA‐specific *cis*‐elements responsible for the ABA‐dependent promoter activation. As the gene/promoter sequences of *TaHDZipI‐5* were not available in databases, we analysed the *TdHDZipI‐5A* and *TdHDZipI‐5B* promoters of homeologous genes from durum wheat. Firstly, several concentrations of ABA were tested to select the minimal endogenous concentration (0.5 mm) leading to a strong promoter activation (data not shown). Secondly, we analysed the 1055‐bp sequence of the *TaHDZipI‐5A* promoter (including 5′UTR), which could activate ABA‐dependent TFs. Mapping revealed that the promoter was activated through the proximal MYBR element GGATA (−310 bp from the translation start), which was identified by Baranowskij *et al*. (1994) as the binding site for MYBSt1, a MYB‐like protein with an endosperm‐related function (Mercy *et al*., 2003). Additionally, the activity of the *TdHDZipI‐5A* promoter was enhanced by two ABREs and/or one MYBR elements, situated upstream of the proximal MYBR element (Figures 2b and S3). All mapped *cis*‐elements were found in the promoters of both homeologous durum genes in conserved positions. Predicted ABRE situated in the D4 fragment of the promoter close to the TATA box was not involved in promoter activation by ABA.

Using a transient expression assay in wheat cells and a reporter construct with synthetic promoter, Harris *et al*. (2016) demonstrated that TaHDZipI‐5 acted as a transcriptional activator. In this work, the transactivation TaHDZipI‐5 domain was defined in an in‐yeast activation assay. A series of CDS deletions encoding truncated variants of TaHDZipI‐5 were generated, and the constructs were transformed in yeast cells to detect transactivation activity. Similar to Zmhdz4 (Wu *et al*., 2016) and Oshox22 (Zhang *et al*., 2012), TaHDZipI‐5 was found to contain an AD at the C‐terminal region of the protein (Figure 3a), which is a highly conserved sequence in homologous proteins from various grasses (Figure 3b).

According to our previous study (Harris *et al*., 2016), all wheat HD‐Zip I γ‐clade members homo‐dimerize and also interact with other members through hetero‐dimerization. We revealed that TaHDZipI‐5 displayed an equal propensity to form homo‐dimeric TaHDZipI‐5 and hetero‐dimeric TaHDZipI‐3/TaHDZipI‐5 complexes. However, the DNA interaction differences between TaHDZipI‐5 homo‐ and hetero‐dimers remained unclear. Hence, we constructed the 3D models of homo‐dimers of TaHDZipI‐5 or TaHDZipI‐3 and hetero‐dimeric TaHDZipI‐3/TaHDZipI‐5, in complex with HDZ1 *cis*‐elements, to explore the differences in DNA binding between homo‐ and hetero‐dimeric structures. 3D models showed that DNA interactions in the TaHDZipI‐3/TaHDZipI‐5 hetero‐dimer were more stable than those in the TaHDZipI‐5 homo‐dimer. These models suggested that the TaHDZipI‐3/TaHDZipI‐5 hetero‐dimer is more efficient in binding DNA. This may indicate that the TaHDZipI‐3/TaHDZipI‐5 hetero‐dimer could be more efficient also *in vivo*, during the activation of target promoters, than the TaHDZipI‐5 homo‐dimer.

Initially, we overexpressed the *TaHDZipI‐5* gene in transgenic wheat plants using a constitutive polyubiquitin promoter from maize. Homozygous T_1_ or T_2_ sublines were selected in a pilot experiment using T_2_ and T_3_ generations, and seeds of selected sublines were used for the analyses of growth characteristics and yield components under sufficient and limited watering (Figure 5). This experiment was performed in large containers to better reflect interplant competition for water, light and nutrients that might occur in the field. We observed that the *TaHDZipI‐5* transgene negatively influenced plant phenotypes by decreasing the numbers of tillers and spikes per plant and consequently decreasing the total plant biomass and seed number compared to those of WT plants. Under well‐watered conditions, the differences in all characteristics were significantly higher than under drought (Figure 5). Differences in flowering time resulted in 1‐ to 3‐day delays in T_3_ sublines compared to the average flowering times of control plants. The differences in flowering times of selected T_1_ plants amounted up to 2 weeks (Figure S6), but these differences decreased in two subsequent generations of transgenic lines. In contrast, the remainder of growth and yield characteristics in the T_1_ generation of transgenic plants diverged less, most probably because they represented a mixture of homo‐ and heterozygous plants, including those of null segregants, which were not excluded from this analysis.

Under well‐watered conditions, lines L2 and L4 had more similar phenotypes compared to those of WT plants and L1 in T_1_ and T_3_ generations. Thus, these two lines were selected for drought tolerance evaluation, which could be defined as plant's ability to survive severe drought at the vegetative developmental stage. Seedlings of transgenic and control plants were grown and assessed in the same pot, to accommodate differences in seedling sizes. These data, using T_2_ plants, suggested approximately fivefold to eightfold enhancement of drought tolerance in both transgenic lines compared to control plants (Figure 6a). This observation is in contradiction with that obtained for *Oshox22*, overexpressed in transgenic rice (Zhang *et al*., 2012), but correlates with the data obtained for *Zmhdz4*, overexpressed in transgenic *Arabidopsis* and rice (Wu *et al*., 2016). For these discrepancies, we offer the following explanation. Both *Zmhdz4* and *TaHDZipI‐5* originate from plants which have different physiological responses to drought compared to rice. Therefore, *Zmhdz4* and *TaHDZipI‐5* may have different biological roles to *Oshox22*, which might be connected to small, but functionally important differences in protein structure or to differences in spatial or temporal patterns of gene expression. These hypotheses require further investigation.

Frost tolerance experiments were performed once with T_1_ transgenic plants (Figure 6b) with null segregants identified and excluded from the analysis and twice using T_3_ homozygous transgenic lines (Figure 6c,d). Both experiments were performed similarly; however, in the second experiment (using T_3_ homozygous transgenic lines), the minimal incubation temperature was 1 °C lower than that in the first experiment (using T_1_ plants). This resulted in a lower survival rate of WT plants in the second experiment. An enhancement of frost tolerance was observed in all tested lines in both experiments, confirming that *TaHDZipI‐5* is a promising candidate gene for improvement of wheat frost tolerance. The analysis of potential downstream stress‐inducible LEA (late embryogenesis abundant)/COR (cold‐responsive)/DHN (dehydrin) genes in control WT and transgenic lines with the constitutive overexpression of the *TaHDZipI‐5* transgene revealed the up‐regulation of *TaCOR14B* (GenBank: AF207546) and *TaRAB15* (GenBank: X59133) transcripts in several transgenic lines (Figure S7). However, this up‐regulation did not correlate with the levels of *TaHDZipI‐5* transgene transcripts (Figure 6d).

Constitutive overexpression of *TaHDZipI‐*5 led to a negative effect of the transgene on the plant phenotype similar to overexpression of *Oshox22* in transgenic rice; this also resulted in a stunted phenotype of transgenic plants, a smaller size of plants and fewer tillers (Zhang *et al*., 2012). To eliminate or decrease the negative effect of the transgene on the phenotype of transgenic wheat lines, the constitutive promoter was replaced with stress‐inducible *pWRKY71* and *pCor39* promoters, which were previously used to optimize the phenotype of transgenic barley (Kovalchuk *et al*., 2013). Both promoters are active in vegetative and flowering parts of the plant. The first promoter originates from the rice *OsWRKY71* gene, and in barley, it was moderately activated by cold and weakly activated by drought. The activity of the promoter was ABA‐independent, and the basal level of activity in transgenic barley was low. In contrast, the second promoter, which originates from the *TdCor39* gene, was strongly activated in barley by frost and drought. *TdCor39* was strongly induced by ABA and had a moderate basal level of activity in barley (Kovalchuk *et al*., 2013).

Transgenic wheat lines with the *TaHDZipI‐*5 transgene driven by *pWRKY71* and *pCor39* promoters showed activation of both promoters under low temperature (Figures 7b and 8b), and low levels of transgene expression in the absence of stress. Inducible transgene expression improved single grain weight compared to when the constitutive polyubiquitin promoter was used. Single grain weights did not decrease in plants with either stress‐inducible promoters compared to WT. Differences in flowering times between transgenic and control plants were smaller in plants with the *pWRKY71* promoter, compared to those containing *pUbi* promoter constructs. However, the improvement or elimination of other negative changes in phenotypes of transgenic lines growing under conditions of sufficient watering was not achieved. This disappointing result was somewhat surprising because the levels of *pWRKY71* and *pCor39* promoter activities in the absence of stress (basal levels) were low. One feasible explanation for these results could be that the application of heterologous promoters led to a ‘poisonous’ effect of the transgene on plant development because of the wrong spatial pattern of *TaHDZipI‐5* expression. This problem could be ameliorated by (i) using the native *TaHDZipI‐5* promoter boosted by the addition of enhancing elements, so that the spatial activity of the promoter remains unchanged (Chen *et al*., 2017), (ii) enhancing the efficiency of translation of the native *TaHDZipI‐5* gene or (iii) manipulation of native TaHDZipI‐5 protein structure to increase the strength of binding to target *cis*‐elements or the efficacy of ABA‐dependent protein activation. These options could be explored with the recently developed technology of genome editing using engineered nucleases, without the generation of transgenic plants with an extra copy of the target gene.

## Experimental procedures

### Plasmid construction and plant transformation

The generation of vectors for plant transformation has been described previously (Eini *et al*., 2013; Kovalchuk *et al*., 2013; Morran *et al*., 2011). Briefly, the *2x35S* promoter was excised from the pMDC32 vector (Curtis and Grossniklaus, 2003) and replaced with one of three promoters: the constitutive *ZmUbiquitin* promoter (Eini *et al*., 2013), or stress‐inducible *OsWRKY71* or *TdCor39* promoters (Kovalchuk *et al*., 2013), resulting in pUbi, pWRKY71 and pCor39 vectors, respectively. A 767‐bp fragment of the *TaHDZipI‐5* coding sequence (CDS) (GenBank accession KT224376) (Harris *et al*., 2016) was isolated from *T. aestivum* L. cv. RAC875 and cloned into a pENTR‐D‐TOPO vector (Invitrogen, Melbourne, Victoria, Australia). The cloned insert was verified by sequencing and subcloned into the pUbi, pWRKY71 and pCor39 vectors, resulting in pUbi‐TaHDZipI‐5, pWRKY71‐TaHDZipI‐5 and pCor39‐TaHDZipI‐5 constructs.

Constructs were transformed in the Australian elite bread wheat cv. Gladius using a biolistic bombardment method (Ismagul *et al*., 2014; Kovalchuk *et al*., 2009). Genomic DNA was isolated from leaf tissue using a freeze‐drying method described by Shavrukov *et al*. (2010). Transgene integration was confirmed by PCR using a forward primer from the 3′ end of the *TaHDZipI‐5* coding region and a reverse primer from the 5′ end of the *nos* terminator (Table S1). Transgene genomic copy number was estimated in the T_0_ and/or T_1_ progenies of selected transgenic lines by quantitative real‐time PCR (Q‐PCR), based on the 2^−ΔΔCt^ method (Kovalchuk *et al*., 2013; Li *et al*., 2004). *Nos* terminator primers and a specific DNA probe (Yadav *et al*., 2015) were used for transgene amplification, and endogenous *Puroindoline‐b* (*Pin‐b*) gene primers and probe (Li *et al*., 2004; Yadav *et al*., 2015) were used for template loading normalization (Table S1). T_1_ lines with a single copy number were selected for further analysis. The selections of homozygous T_1_ lines were conducted in pilot experiments using the T_2_ progeny of transgenic T_1_ lines with two copies of transgene or T_3_ progeny of T_2_ lines. In these experiments, transgene integration was assessed by PCR in twelve seedlings of each line. The line was considered as homozygous if the expected PCR product was observed for all twelve plants. Seeds of homozygous T_1_ (or T_2_) lines from each construct were selected and used for phenotyping and stress tolerance tests.

Appendix S1 contain the descriptions of gene expression by quantitative real‐time PCR, cloning of promoters and the identification of ABA‐responsive *cis*‐elements, analysis of evolutionary relationships, in‐yeast activation assay, construction of 3D models, analysis of transgenic plants, drought tolerance tests and survival rates of seedlings under terminal drought, and frost tolerance tests.

## Conflict of interest

The authors declare no conflict of interest.

## Supporting information


**Figure S1** Soil water tension monitored at 10 and 30 cm depths in large containers used for plant growth under well‐watered conditions or increasing drought.
**Figure S2** Details of frost tolerance experiments.
**Figure S3** Alignments of *TdHDZipI‐5A* and *TdHDZipI‐5B* promoter sequences and sequences of corresponding genes of *Triticum aestivum* cv. Chinese Spring, identified in the Whole Genome Reference Assembly Pseudomolecules v1.0 databases of the International Wheat Genome Sequencing Consortium, using the BLAST software (Altschul *et al*., 1997).
**Figure S4** Alignment of *TdHDZipI‐5B* (5B) and *TdHDZipI‐5A* (5A) promoters. LALIGN (Huang and Miller, 1991) was used to find the best local alignments.
**Figure S5** (a) Transgene copy numbers in T_1_ transgenic plants estimated by Q‐PCR. Plants seeds used in analyses are indicated by arrows. (b) Examples of selection of homozygous lines by PCR using transgene‐specific primers.
**Figure S6** Growth characteristics and yield components of control wild‐type (WT) and transgenic T_1_ wheat (*Triticum aestivum* cv. Gladius) plants transformed with pUbi‐TaHDZipI‐5.
**Figure S7** Expression levels of three stress‐inducible LEA (Late Embryogenesis Abundant)/COR (cold‐responsive)/DHN (dehydrin) genes (*TaWZY2*, GenBank: EU395844; *TaCOR14B*, GenBank: AF207546; *TaRab15*, GenBank: X59133) and the TaDREB3 (GenBank: DQ353853) regulatory gene, in leaves of unstressed control WT plants and T_3_ sublines of tree independent transgenic lines.
**Table S1** List of PCR primers and DNA probes used in this study.
**Table S2** A sequence alignment of 14 entries (with GenBank accession numbers) used to generate a phylogenetic tree displaying the evolutionary relationships of HD‐Zip I γ‐clade TFs from *Arabidopsis* and selected monocots, shown in Figure 1.
**Table S3** Hydrogen bonds of homo‐dimeric TaHDZipI‐3 and TaHDZipI‐5, and hetero‐dimeric TaHDZipI‐3/TaHDZipI‐5 with HDZ1 (5′‐CAATCATTGC‐3′/5′‐GCAATGATTG‐3′).
**Table S4** Characteristics of the T_2_/T_3_ progenies of *TaHDZipI‐5* transgenic lines analysed in large containers under well‐watered or mild drought condition.
**Appendix S1** Materials and methods.
