# Supplementary material for: Overexpression of the class I homeodomain transcription factor TaHDZipI‐5 increases drought and frost tolerance in transgenic wheat
Source: Plant Biotechnol J. 2017 Dec 27;16(6):1227–40. doi: 10.1111/pbi.12865 (PMC5978581; doi:10.1111/pbi.12865)
Supplement: Supplementary file 1 — Figure S1 Soil water tension monitored at 10 and 30 cm depths in large containers used for plant growth under well‐watered conditions or increasing drought. Figure S2 Details of frost tolerance experiments. Figure S3 Alignments of TdHDZipI‐5A and TdHDZipI‐5B promoter sequences and sequences of corresponding genes of Triticum aestivum cv. Chinese Spring, identified in the Whole Genome Reference Assembly Pseudomolecules v1.0 databases of the International Wheat Genome Sequencing Consortium, using the BLAST software (Altschul et al., 1997). Figure S4 Alignment of TdHDZipI‐5B (5B) and TdHDZipI‐5A (5A) promoters. LALIGN (Huang and Miller, 1991) was used to find the best local alignments. Figure S5 (a) Transgene copy numbers in T1 transgenic plants estimated by Q‐PCR. Plants seeds used in analyses are indicated by arrows. (b) Examples of selection of homozygous lines by PCR using transgene‐specific primers. Figure S6 Growth characteristics and yield components of control wild‐type (WT) and transgenic T1 wheat (Triticum aestivum cv. Gladius) plants transformed with pUbi‐TaHDZipI‐5. Figure S7 Expression levels of three stress‐inducible LEA (Late Embryogenesis Abundant)/COR (cold‐responsive)/DHN (dehydrin) genes (TaWZY2, GenBank: EU395844; TaCOR14B, GenBank: AF207546; TaRab15, GenBank: X59133) and the TaDREB3 (GenBank: DQ353853) regulatory gene, in leaves of unstressed control WT plants and T3 sublines of tree independent transgenic lines. Table S1 List of PCR primers and DNA probes used in this study. Table S2 A sequence alignment of 14 entries (with GenBank accession numbers) used to generate a phylogenetic tree displaying the evolutionary relationships of HD‐Zip I γ‐clade TFs from Arabidopsis and selected monocots, shown in Figure 1. Table S3 Hydrogen bonds of homo‐dimeric TaHDZipI‐3 and TaHDZipI‐5, and hetero‐dimeric TaHDZipI‐3/TaHDZipI‐5 with HDZ1 (5′‐CAATCATTGC‐3′/5′‐GCAATGATTG‐3′). Table S4 Characteristics of the T2/T3 progenies of TaHDZipI‐5 transgenic lines analysed [file PBI-16-1227-s001.pdf]

Overexpression of the class I homeodomain transcription factor TaHDZipI-5 increases drought and frost tolerance in transgenic wheat

Yunfei Yang, Sukanya Luang[†], John Harris[††], Matteo Riboni, Yuan Li, Natalia Bazanova[‡], Maria Hrmova*, Stephan Haefele[‡‡], Nataliya Kovalchuk, Sergiy Lopato

University of Adelaide, School of Agriculture, Food and Wine, Glen Osmond, SA 5064, Australia

*Correspondence: maria.hrmova@adelaide.edu.au; Tel: +61 8 8313 7160; Fax: +61 8 8313 7102

Current addresses:

[†]Institute of Molecular Biosciences, Mahidol University, Nakhon-Pathom, Thailand

††South Australian Research and Development Institute, GPO Box 397, Adelaide, SA 5064, Australia

[‡]Commonwealth Scientific and Industrial Research Organisation, Glen Osmond, SA 5064, Australia

[‡‡]Rothamsted Research, West Common Harpenden, Hertfordshire, Al5 2JQ, UK

Supplementary data

Supplementary materials and methods

Analysis of gene expression by quantitative real-time PCR (*Q-PCR*)

Expression of the transgene in unstressed and cold (4°C) treated leaves of transgenic plants was demonstrated in different generations by Q-PCR (Fletcher, 2014). Total RNA was extracted

from tissues using TRIzol® Reagent (Life Technologies Corporation, Grand Island, NY, USA). Complementary DNA (cDNA) was synthesized from RNA as a template *via* reverse transcription (Fletcher, 2014). cDNA quality was assessed by reverse transcription polymerase chain reaction (RT-PCR) with intron-spanning primers of the wheat house-keeping gene *calreticulin* (*TaCRT*) (Table S1). To determinate the transgene expression level, Q-PCR was performed with primers of the target gene *TaHDZipI-5* (Table S1), described by Ferdous *et al.* (2015). Three out of four optimised wheat house-keeping genes, *TaActin*, *TaCyclophilin*, *TaGAPdH* and *TaEFA*, were used for transgene expression normalisation (Table S1). All experiments were carried out with three biological and three technical replicates.

Cloning of TdHDZipI-5A *and* TdHDZipI-5B *promoters and the identification of abscisic acid* (*ABA*)*-responsive* cis-*elements*

A fragment of the coding region of *TaHDZipI-5* (GeneBank accession KT224376) was isolated by PCR, using full-length cDNA as a template. It was used as a probe to screen a BAC library prepared from genomic DNA of *Triticum turgidum L. ssp. durum cv. Langdon* (Cenci *et al.*, 2003), as described by Kovalchuk *et al*. (2009). Both durum wheat homeologues of the *TaHDZipI-5* gene were identified by PCR using DNA of selected BAC clones as templates, and primers were derived from the coding region of *TaHDZipI-5* cDNA. Genes of the *T. turgidum* ssp *durum*, orthologues of *TaHDZipI-5*, are designated as *TdHDZipI-5A* and *TdHDZipI-5B*. The promoter sequences were identified through sequencing of BAC clones as described by Kovalchuk *et al*. (2009). Approximately 1300-bp-long fragments of the *TdHDZipI-5* promoter sequences were used for promoter analyses. These were cloned into the pENTR-D-TOPO vector, verified by sequencing and re-cloned into pMDC164 (Curtis and Grossniklaus, 2003) upstream of the *GUS* reporter gene. Sequences of the promoters were aligned using LALIGN version 2.1u09 (Huang and Miller, 1991). Potential ABA responsive

cis-elements of promoters were predicted (PLACE software; Higo *et al.*, 1999). Forward primers (Table S1) were designed to prevent interruptions of potential ABA responsive *cis*-elements; four 5'-deletions of the *TdHDZipI-5A* promoter, which included 5'UTRs, were generated by PCR. PCR products were cloned into the pENTR-D-TOPO vector (Invitrogen, Melbourne, Victoria, Australia), confirmed by sequencing and transferred by recombination into pMDC164; these were designated as *TdHDZipI-5A* D1, (1,055 bp), D2 (366 bp), D3 (336 bp), and D4 (175 bp), respectively. The identification of functional *cis*-elements responsible for ABA-induced activation of the *TdHDZipI-5A* promoter by deletions, was performed using a transient expression assay based on the biolistic bombardment of cultured wheat cells (Eini *et al.*, 2013). After one hour of cell recovery following bombardment, the liquid medium was exchanged for the same medium containing 0.5 mM ABA. Blue GUS foci were numbered after 24 hours of incubation with ABA.

Analysis of evolutionary relationship of selected HD-Zip I γ-clade proteins

HD-Zip I γ-clade protein sequences from *Arabidopsis* (Henriksson *et al.*, 2005) and selected monocots (Agalou *et al.*, 2008; Harris *et al.*, 2016; Hu *et al.*, 2012; Zhao *et al.*, 2011) were derived from the Plant Transcription Factor Database (Jin *et al.*, 2017) and EST database at National Center for Biotechnology Information. Multiple sequence alignments (Table S2) were performed using the MAFFT version 7 algorithm online (Katoh and Standley, 2013). A phylogenetic tree was constructed with the Neighbour Joining (NJ) algorithm, p-distance and the bootstrap method (1000 replicates of bootstrap) in the Molecular Evolutionary Genetics Analysis version 6 (MEGA6) program (Tamura *et al.*, 2013).

In-yeast activation assay

A transactivation assay in yeast was used to identify the activation domain of TaHDZipI-5. The full-length open reading frame (ORF) or various truncated fragments of *TaHDZipI-5* were individually fused in frame with the yeast GAL4 DNA-binding domain in the pGBKT7 vector (Invitrogen, Victoria, Australia). Constructs were transformed into yeast (*Saccharomyces cerevisiae* strain AH109). Transformed yeast cells were examined on synthetic defined (SD) (-Trp) medium and replica-plated to SD (-Trp / -His) medium. Yeast growth on the SD medium reflected the growth of yeast containing the native activation domain in truncated TaHDZipI-5 sequences.

Construction of 3D models of homeodomains (HDs) of TaHDZipI-5 in homo- and hetero-dimeric forms in complex with the HDZ1 cis-*element*

Structural models of homo-dimeric TaHDZipI-5 HDs and hetero-dimeric TaHDZipI-3/TaHDZipI-5 HDs were constructed using the crystal structure of *Drosophila melanogaster* HD (PDB: 1JGG) as a template (Hirsch and Aggarwal, 1995), through the MODELLER program suite v9.16 (Sali and Blundell, 1993). A sequence alignment between TaHDZipI-3, TaHDZipI-5 and the template was performed with MUSCLE (Edgar, 2004) and visualised in Annotator (Gille *et al.*, 2014). The DNA *cis*-element HDZ1 (5'-CAATCATTGC-3'/5'-GCAATGATTG-3') was constructed from the AT-rich *cis*-element (5'-TAATTGAATT-3'/5'-AATTCAATTA-3') of 1JGG using Coot (Emsley *et al.*, 2010). Fifty models were generated using different random starting coordinates, and models with the lowest score of the Modeller Objective Function (MOF) (Shen and Sali, 2006) and Discrete Optimised Protein Energy (DOPE) (Eswar *et al.*, 2006) were selected. The final protein models were chosen based on conformational energy calculations with ProSa2003 (Sippl, 1993) that were validated by PROCHECK (Laskowski *et al.*, 1993). DOPE/MOF/z-score parameters for TaHDZipI-5/HDZ1 and TaHDZipI-3/TaHDZipI-5/HDZ1 were -9,716/898/-4.45 and -10,253/810/-6.30,

respectively. The construction of structural models of homo-dimeric TaHDZipI-3 HDs was described previously (Harris *et al.*, 2016). The stabilities of 3D models were calculated using FoldX (Schymkowitz *et al*., 2005).

Analysis of transgenic plants

Three independent T_1 lines of transgenic wheat with a single copy of the transgene were selected for primary phenotypic characterisation and seed multiplication. Twelve seeds of control plants (WT) and twelve T_1 transgenic seeds from each line were sown into 12-cm square pots filled with coco-peat soil, with one plant per pot, and grown under well-watered conditions in a greenhouse with day/night temperatures of 23°C (16 hours) and 19°C (8 hours). Leaves of three-week old control and transgenic seedlings of each line were sampled for genomic DNA isolation. Plant height, tiller and spike number, seed weight, total dry biomass, seed number, flowering time and single grain weight were recorded for each plant. The transgene copy number was determined by Q-PCR (Fig. S1).

Comparative evaluations of growth and yield components of transgenic T_3 lines and control plants grown under well-watered and mild-drought conditions were performed in two large containers filled with a mixture of coco-peat, sand and clay soil (1:1:1) (Shavrukov *et al.*, 2016). Three independent T_3 lines of all four transgenics were used for the evaluation of growth and yield components. Untransformed WT plants were used as control. Transgenic plants were grown in two identical containers, one with well-watered conditions and one with slowly increasing drought. In each container, 16 plants of each transgenic line and the same number of WT plants were randomly grown in rows, with eight plants per row. Leaf samples of each plant were collected for DNA/RNA isolation at the three-leaf stage of seedling development. In the well-watered container, plants were regularly watered until maturity. In the drought-

subjected container, plants were regularly watered until mid-tillering and watering stopped thereafter. Plants showed signs of mild wilting at the beginning of flowering. The soil water potential of each container was automatically monitored and recorded by Magpie-3 (Measuring Engineering Australia) using sensors in two depths (10 cm and 30 cm) below the soil surface (Fig. S1). Growth and yield characteristics of transgenic lines and control plants were monitored in both containers. The data for each measured parameter for each line were statistically analysed using Student *t-tests* (unpaired, two-tails), and null-segregants were excluded from the analyses in cases where lines were heterozygous.

Drought tolerance test or the survival rate of seedlings under terminal drought

Two independent homozygous lines with minimal differences in a seedling size to those of control plants were used in a drought survival test, conducted in a PC2 glasshouse. WT plants were used as control. Seeds were sown in five 6-inch round pots filled with the same amount of coco-peat soil. Before sowing seeds, the soil in each pot was water-saturated by soaking the pot in water overnight in plastic trays. The following day, pots were removed and drained for 24 hours, and each pot was weighed after drainage. The soil moisture weight was calculated as the difference between the soil weight after drainage and the dry soil weight (measured after incubation for a week at 65°C). Two plants of each line and WT plants were grown in each pot in the growth room under 24°C during 16 hours day light and 19°C during 8 hours darkness. Plants in each pot were well-watered for three weeks, after which watering was stopped. During the well-watered stage, each pot was weighted daily and water was added if the soil water content was below 80% of soil moisture weight. After 25 to 28 days of drought, plants were re-watered and survival rates were assessed after a three-week recovery.

Frost tolerance test (survival rate of seedlings subjected to frost)

Three T_3 independent homozygous lines of transgenic plants were used in a frost survival test. Untransformed WT plants were used as control. Seeds were sown in twelve 6-inch round pots filled with coco-peat soil. One plant of each line and WT plants were grown in a pot (Fig. S2A). Plants in each pot were well-watered and kept in a PC2 room (24/16 ^{o}C of day/night temperature, 16 hours day length) for three weeks and later placed into a cold cabinet (BINDER, Tuttlingen, Germany). Plants in the cold cabinet were exposed to temperatures decreasing gradually from 18 ^{o}C to a minimum temperature of -8 ^{o}C with 6.5 hours under the lowest temperature, and then slowly returned back to 18 ^{o}C (Fig. S2B). Pots were insulated to protect plant roots from frost-damage. Leaf tissues for RNA isolation were collected before the stress application (Fig. S2B). In addition, leaves of plants with stress-inducible promoters and control plants were collected at 4 ^{o}C. The ice nucleating agent SNOMAX® (Sno-Quip Pty Ltd, Mittagong, NSW, Australia) (2 g/L) was used to spray plants to prevent water crystallisation below 0 ^{o}C. After the frost treatment, pots were transferred back to the PC2 growth room for recovery. Survival rates were estimated after two weeks of recovery.

Analysis of expression of potential downstream genes in transgenic lines with constitutive overexpression of TaHDZipI-5

The analysis of the downstream gene expression was performed by Q-PCR, as described by Fletcher *et al*. (2014). Gene-specific primers from 3'UTRs (Table S1) were used to analyse the expression levels of *TaWZY2* (GenBank: EU395844), *TaCOR14B* (GenBank: AF207546; Tsvetanov *et al*., 2000), *TaRAB15* (GenBank: X59133; King *et al*., 1992) and *TaDREB3* (GenBank: DQ353853; Lopato *et al*., 2006) genes in three independent control WT plants and three T_3 sublines of each of three independent transgenic lines with the constitutive overexpression of *TaHDZipI-5*. Three technical replicates were used in this experiment.

Legends to supplementary figures

Fig. S1. Soil water tension monitored at 10 cm and 30 cm depths in large containers used for plant growth under well-watered conditions or increasing drought. An arrow (no watering) indicates the point at which watering was withdrawn.

Fig. S2. Details of frost tolerance experiments. (a) Position of seedlings in pots during frost tolerance tests. (b) Temperature and light conditions during frost tolerance experiments in a semiautomatic cold cabinet.

Fig. S3. Alignments of *TdHDZipI-5A* and *TdHDZipI-5B* promoter sequences and sequences of corresponding genes of *Triticum aestivum* cv. Chinese Spring, identified in the Whole Genome Reference Assembly Pseudomolecules v1.0 databases of the International Wheat Genome Sequencing Consortium, using the BLAST software (Altschul *et al.*, 1997).

Fig. S4. Alignment of *TdHDZipI-5B* (5B) and *TdHDZipI-5A* (5A) promoters. LALIGN (Huang and Miller, 1991) was used to find the best local alignments. Primers used for generation of promoter deletions are underlined. Characteristic elements present in sequences of both promoters are indicated with boxes of different colours. MYBR - MYB recognition element; ABRE - abscisic acid responsive element; ATG - translational start.

Fig. S5. (a) Transgene copy numbers in T_1 transgenic plants estimated by Q-PCR. Plants seeds used in analyses are indicated by arrows. (b) Examples of selection of homozygous lines by PCR using transgene-specific primers. Homozygous T_1 sublines of two independent single-transformation-event lines were selected using the analysis of the transgene (*TaHDZipI-5*) presence in the T_2 progeny. H_2O - sample containing no DNA, WT - sample containing DNA

isolated from WT untransformed plant, P - positive control, where 1000-fold diluted plasmid DNA was used as a template.

Fig. S6. Growth characteristics and yield components of control wild-type (WT) and transgenic T_1 wheat (*Triticum aestivum* cv. Gladius) plants transformed with pUbi-TaHDZipI-5. Plants were grown under well-watered conditions. Flowering time of transgenic plants was compared with the average flowering time of 12 control WT plants, which is represented as day 0. Differences between transgenic lines and WT plants were tested in the unpaired Student's t-test (* $P<0.05$, ** $P<0.01$, *** for $P < 0.001$).

Fig. S7. Expression levels of three stress-inducible LEA (Late Embryogenesis Abundant)/COR (Cold-Responsive)/DHN (Dehydrin) genes (*TaWZY2*, GenBank: EU395844; *TaCOR14B*, GenBank: AF207546; *TaRab15*, GenBank: X59133) and the TaDREB3 (GenBank: DQ353853) regulatory gene, in leaves of unstressed control WT plants and T_3 sublines of tree independent transgenic lines. Expression levels of the *TaHDZipI-5* transgene in the same lines are shown in Fig. 6D. No correlation was found between the expression levels of the *TaHDZipI-5* transgene and downstream genes. Error bars represent ± SD of three technical replicates.

Legends to supplementary tables

Table S1. List of PCR primers and DNA probes used in this study.

Table S2. A sequence alignment of 14 entries (with GenBank accession numbers) used to generate a phylogenetic tree displaying the evolutionary relationships of HD-Zip I γ-clade TFs from *Arabidopsis* and selected monocots, shown in Fig. 1. Asterisks (*) indicate positions with a single conserved residue; colons (:) indicate conservation between residues with strongly

similar properties (scoring > 0.5 in the Gonnet PAM 250 matrix); full stops (.) indicate conservation between residues with weakly similar properties (scoring ≤ 0.5 in the Gonnet PAM 250 matrix).

Table S3. Hydrogen bonds of homo-dimeric TaHDZipI-3 and TaHDZipI-5, and hetero-dimeric TaHDZipI-3/TaHDZipI-5 with HDZ1 (5'-CAATCATTGC-3'/5'-GCAATGATTG-3').

Table S4. Characteristics of the T_2/T_3 progenies of *TaHDZipI-5* transgenic lines analysed in large containers under well-watered or mild drought condition.

Supplementary references

Agalou, A., Purwantomo, S., Overnas, E., Johannesson, H., Zhu, X., Estiati, A., Kam, R.J.D. *et al.* (2008) A genome-wide survey of HD-Zip genes in rice and analysis of drought-responsive family members. *Plant Mol. Biol.* **66**, 87–103.

Altschul, S. F., Madden, T. L., Schaeffer, A. A., Zhang, J., Zhang, Z., Miller, W. and Lipman D. J. (1997) Gapped BLAST and PSI-BLAST: a new generation of protein database search programs. *Nucleic Acids Res.* **25**, 3389–3402.

Cenci, A., Chantret, N., Kong, X., Gu, Y., Anderson, O.D., Fahima, T., Distelfeld, A. *et al.* (2003). Construction and characterization of a half million clone BAC library of durum wheat (*Triticum turgidum* ssp *durum*). *Theor. Applied Genet.* **107**, 931–939.

Curtis, M.D. and Grossniklaus, U. (2003) A gateway cloning vector set for high-throughput functional analysis of genes in planta. *Plant Physiol.* **133**, 462–469.

Edgar, R.C. (2004) MUSCLE: multiple sequence alignment with high accuracy and high throughput. *Nucleic Acids Res.* **32**: 1792–1797.

Eini, O., Yang, N., Pyvovarenko, T., Pillman, K., Bazanova, N., Tikhomirov, N., Eliby, S. *et al.* (2013) Complex regulation by Apetala2 domain-containing transcription factors revealed through analysis of the stress-responsive *TdCor410b* promoter from durum wheat. *PloS One* **8**, e58713.

Emsley, P., Lohkamp, B., Scott, W.G. and Cowtan, K. (2010) Features and development of Coot. *Acta Crystallogr.* **66**, 486–501.

Eswar, N., Webb, B., Marti-Renom, M.A., Madhusudhan, M.S., Eramian, D., Shen, M.-Y., Pieper, U. *et al.* (2006) Comparative protein structure modeling using Modeller. *Curr. Protoc. Bioinform.*: John Wiley & Sons, Inc.

Ferdous, J., Li, Y., Reid, N., Langridge, P., Shi, B.J. and Tricker, P.J. (2015) Identification of reference genes for quantitative expression analysis of microRNAs and mRNAs in barley under various stress conditions. *PloS One* **10**, e0118503.

Fletcher, S.J. (2014) qPCR for quantification of transgene expression and determination of transgene copy number. *Meth. Mol. Biol.* **1145**, 213–237.

Gille, C., Fähling, M., Weyand, B., Wieland, T. and Gille, A. (2014) Alignment-annotator web server: rendering and annotating sequence alignments. *Nucleic Acids Res.* 42, W3-W6.

Harris, J.C., Sornaraj, P., Taylor, M., Bazanova, N., Baumann, U., Lovell, B., Langridge, P. *et al.* (2016) Molecular interactions of the γ-clade homeodomain-leucine zipper class I transcription factors during the wheat response to water deficit. *Plant Mol. Biol.* **90**, 435–452.

Henriksson, E., Olsson, A.S., Johannesson, H., Johansson, H., Hanson, J., Engström, P. and Söderman, E. (2005) Homeodomain leucine zipper class I genes in *Arabidopsis*. Expression patterns and phylogenetic relationships. *Plant Physiol.* **139**, 509–518.

Higo, K., Ugawa, Y., Iwamoto, M. and Korenaga, T. (1999) Plant *cis*-acting regulatory DNA elements (PLACE) database: 1999. *Nucleic Acids Res.* **27**, 297–300.

Hirsch, J.A. and Aggarwal, A.K. (1995) Structure of the even-skipped homeodomain complexed to AT-rich DNA: new perspectives on homeodomain specificity. *EMBO J.* **14**, 6280-6291.

Hu, R., Chi, X., Chai, G., Kong, Y., He, G., Wang, X., Shi, D. *et al.* (2012) Genome-wide identification, evolutionary expansion, and expression profile of homeodomain-leucine zipper gene family in poplar (*Populus trichocarpa*). *PloS One* **7**, e31149.

Huang, X. and Miller, W. (1991) A time-efficient, linear-space local similarity algorithm. *Adv. Appl. Math.* **12**, 337–357.

Jin, J., Tian, F., Yang, D.C., Meng, Y.Q., Kong, L., Luo, J. and Gao, G. (2017) PlantTFDB 4.0: toward a central hub for transcription factors and regulatory interactions in plants. *Nucleic Acids Res.* **45**, D1040–D1045.

Katoh, K. and Standley, D.M. (2013) MAFFT multiple sequence alignment software version 7: improvements in performance and usability. *Mol. Biol. Evol.* **30**, 772–780.

King, S.W., Joshi, C. and Nguyen, H.T. (1992) DNA sequence of an ABA-responsive gene (*rab15*) from water-stressed wheat roots. *Plant Mol. Biol.* **18**, 119–121.

Laskowski, R.A., MacArthur, M.W., Moss, D.S. and Thornton, J.M. (1993) PROCHECK: a program to check the stereochemical quality of protein structures. *J. Appl. Crystallogr.* **26**, 283–291.

Lopato, S., Bazanova, N., Morran, S., Milligan, A.S., Shirley, N. and Langridge, P. (2006) Isolation of plant transcription factors using a modified yeast one-hybrid system. *Plant Meth.* **2**, 3–17.

Sali, A. and Blundell, T. (1993) Comparative protein modeling by satisfaction of spatial restraints. *J. Mol. Biol.* **234**, 779–815.

Schymkowitz, J.W., Rousseau, F., Martins, I.C., Ferkinghoff-Borg, J., Stricher, F. and Serrano, L. (2005) Prediction of water and metal binding sites and their affinities by using the Fold-X force field. *Proc. Natl. Acad. Sci. USA* **102**, 10147–10152.

Shavrukov, Y., Baho, M., Lopato, S. and Langridge, P. (2016) The *TaDREB3* transgene transferred by conventional crossings to different genetic backgrounds of bread wheat improves drought tolerance. *Plant Biotechnol. J.* **14**, 313–322.

Shen, M., Sali, A. (2006) Statistical potential for assessment and prediction of protein structures. *Prot. Sci.* **15**, 2507–2524.

Sippl, M.J. (1993) Recognition of errors in three-dimensional structures of proteins. *Proteins: Struct. Funct. Bioinf.* **17**, 355–362.

Tamura, K., Stecher, G., Peterson, D., Filipski, A. and Kumar, S. (2013) MEGA6: molecular evolutionary genetics analysis version 6.0. *Mol. Biol. Evol.* **30**, 2725–2729.

Tsvetanov, S., Ohno, R., Tsuda, K., Takumi, S., Mori, N., Atanassov, A. and Nakamura, C. (2000) A cold-responsive wheat (*Triticum aestivum* L.) gene *wcor14* identified in a winter-hardy cultivar Mironovska 808. *Genes Genet. Syst.* **75**, 49–57.

Zhao, Y., Zhou, Y., Jiang, H., Li, X., Gan, D., Peng, X., Zhu, S. *et al.* (2011) Systematic analysis of sequences and expression patterns of drought-responsive members of the *HD-Zip* gene family in maize. *PloS One* **6**, e28488.

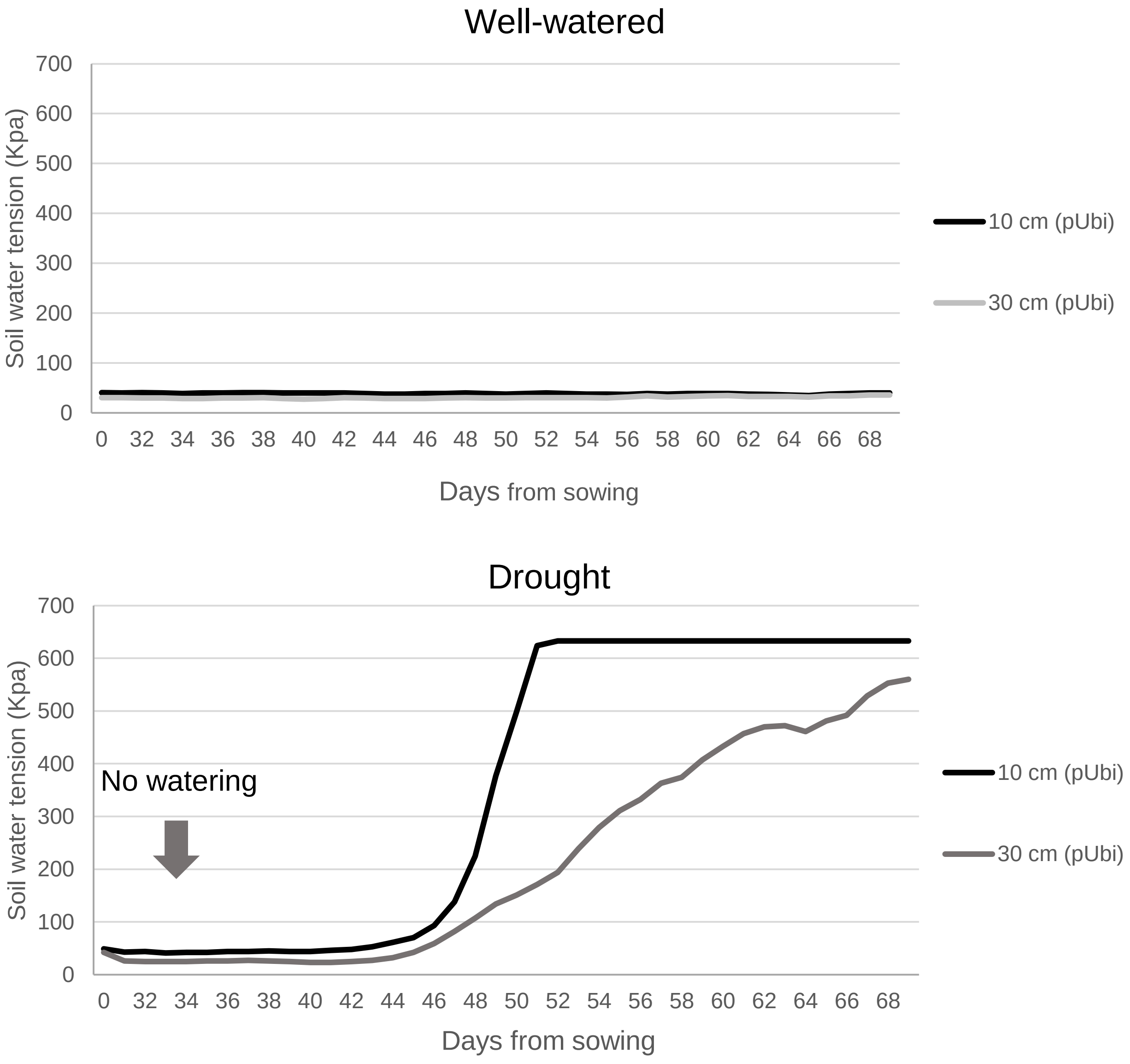

Fig. S1. Soil water tension monitored at 10 cm and 30 cm depths in large containers used for plant growth under well-watered conditions or increasing drought. An arrow (no watering) indicates the point at which watering was withdrawn.

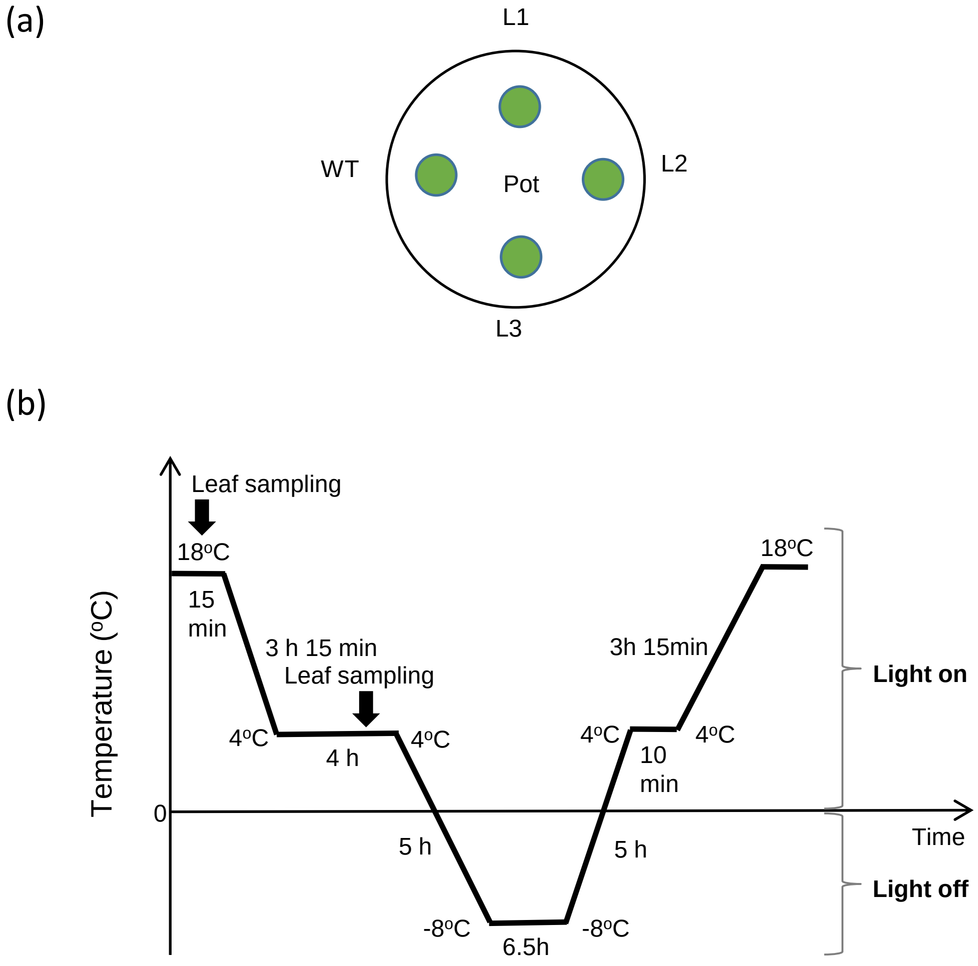

Fig. S2. Details of frost tolerance experiments. (a) Position of seedlings in pots during frost tolerance tests. (b) Temperature and light conditions during frost tolerance experiments in a semi-automated cold cabinet.

```
Query= TdHDZipI-5A (promoter + 5'UTR sequence) (2354 letters)
Sequences producing significant alignments: chr2B (Sbjct)
Score = 2409 bits (1215), Expect = 0.0
Identities = 1218/1219 (99%)
Strand = Plus / Plus

Query: 1136      gtatcgtgctactgcggctcgaaatgacctgacccccgccgttaccgaagtcggaagaac 1195
                 ||||||||||||||||||||||||||||||||||||||||||||||||||||||||||||
Sbjct: 578603883 gtatcgtgctactgcggctcgaaatgacctgacccccgccgttaccgaagtcggaagaac 578603942

Query: 1196      tccaccagcgcactagcattcaacaaataaaggcagttcagtcgcaccgggtcaccgctt 1255
                 ||||||||||||||||||||||||||||||||||||||||||||||||||||||||||||
Sbjct: 578603943 tccaccagcgcactagcattcaacaaataaaggcagttcagtcgcaccgggtcaccgctt 578604002

Query: 1256      ttaacttacctgatctgctctagcacgtttggatcgtgggaggtcgggggcagcgaatcg 1315
                 ||||||||||||||||||||||||||||||||||||||||||||||||||||||||||||
Sbjct: 578604003 ttaacttacctgatctgctctagcacgtttggatcgtgggaggtcgggggcagcgaatcg 578604062

Query: 1316      ccggcctccgggtggaaaacgatcgatcgaggataacctctgctccccgcactgacaccc 1375
                 ||||||||||||||||||||||||||||||||||||||||||||||||||||||||||||
Sbjct: 578604063 ccggcctccgggtggaaaacgatcgatcgaggataacctctgctccccgcactgacaccc 578604122

Query: 1376      ccaagtgctcgcctatcacgttcacttcaccgtatctatctatcctgagttaccatgatg 1435
                 ||||||||||||||||||||||||||||||||||||||||||||||||||||||||||||
Sbjct: 578604123 ccaagtgctcgcctatcacgttcacttcaccgtatctatctatcctgagttaccatgatg 578604182

Query: 1436      tctcttcctggtaaacgatcgatggagtaaactagtggagcgcaccaggacaacaggcga 1495
                 ||||||||||||||||||||||||||||||||||||||||||||||||||||||||||||
Sbjct: 578604183 tctcttcctggtaaacgatcgatggagtaaactagtggagcgcaccaggacaacaggcga 578604242

Query: 1496      caagaccggccaacgcgcgcggcgcgacgcgctagctatagtgtgctctgtgcatcggcc 1555
                 ||||||||||||||||||||||||||||||||||||||||||||||||||||||||||||
Sbjct: 578604243 caagaccggccaacgcgcgcggcgcgacgcgctagctatagtgtgctctgtgcatcggcc 578604302

Query: 1556      cggtcgttggagcccgggaacgggacaaacgcggcgcgccacctcgacgccccttccggc 1615
                 ||||||||| ||||||||||||||||||||||||||||||||||||||||||||||||||
Sbjct: 578604303 cggtcgttgcagcccgggaacgggacaaacgcggcgcgccacctcgacgccccttccggc 578604362

Query: 1616      ggaagcccccgcacacctggcgccgcgacggcgacgcgtgcagcccccgacggagctagg 1675
                 ||||||||||||||||||||||||||||||||||||||||||||||||||||||||||||
Sbjct: 578604363 ggaagcccccgcacacctggcgccgcgacggcgacgcgtgcagcccccgacggagctagg 578604422

Query: 1676      ccctctgggcgcgcacggccattggtcctacctgttcctcctcggcccgtccccgtctcc 1735
                 ||||||||||||||||||||||||||||||||||||||||||||||||||||||||||||
Sbjct: 578604423 ccctctgggcgcgcacggccattggtcctacctgttcctcctcggcccgtccccgtctcc 578604482

Query: 1736      ccgtggaacaccaaggcgcacacctttccaccacccattggccggcaagccccatgatac 1795
                 ||||||||||||||||||||||||||||||||||||||||||||||||||||||||||||
Sbjct: 578604483 ccgtggaacaccaaggcgcacacctttccaccacccattggccggcaagccccatgatac 578604542

Query: 1796      actactgaggactggggtactgctgatcctttctcgtgcagctgatcgatcgattcgtag 1855
                 ||||||||||||||||||||||||||||||||||||||||||||||||||||||||||||
Sbjct: 578604543 actactgaggactggggtactgctgatcctttctcgtgcagctgatcgatcgattcgtag 578604602

Query: 1856      aatcgtagtacatgaaaatgcgagaagtggacagacagagaagaaaataaacgaaaagaa 1915
                 ||||||||||||||||||||||||||||||||||||||||||||||||||||||||||||
Sbjct: 578604603 aatcgtagtacatgaaaatgcgagaagtggacagacagagaagaaaataaacgaaaagaa 578604662

Query: 1916      cacgtccggtcgacttaatttgcaattcgtaggccggaaggaagacttgaatgagcagga 1975
                 ||||||||||||||||||||||||||||||||||||||||||||||||||||||||||||
Sbjct: 578604663 cacgtccggtcgacttaatttgcaattcgtaggccggaaggaagacttgaatgagcagga 578604722

Query: 1976      ggaggaggagaagcaagatctttgcactcgtggccacgtttcgggtgccgatctcgaggc 2035
                 ||||||||||||||||||||||||||||||||||||||||||||||||||||||||||||
Sbjct: 578604723 ggaggaggagaagcaagatctttgcactcgtggccacgtttcgggtgccgatctcgaggc 578604782

Query: 2036      tggaggatagacatgggggcgccgtggtgggcacagcttctggcgccgctggatgcgcga 2095
                 ||||||||||||||||||||||||||||||||||||||||||||||||||||||||||||
Sbjct: 578604783 tggaggatagacatgggggcgccgtggtgggcacagcttctggcgccgctggatgcgcga 578604842

Query: 2096      cacttggcctggcggacgaaagcggctttgcgcggtgtcaggctgcgtgggtcccgcccg 2155
                 ||||||||||||||||||||||||||||||||||||||||||||||||||||||||||||
Sbjct: 578604843 cacttggcctggcggacgaaagcggctttgcgcggtgtcaggctgcgtgggtcccgcccg 5786049

Query: 2156      ggaggtttctttatccgccatcgcgaagaccacatgttcgcatctcgcgcaacgcggaca 2215
                 ||||||||||||||||||||||||||||||||||||||||||||||||||||||||||||
Sbjct: 578604903 ggaggtttctttatccgccatcgcgaagaccacatgttcgcatctcgcgcaacgcggaca 578604962
```

```
Query: 2216      cgtaacgccgtccgccggtataaaaccggcctcccggcgccgagagacagaggaagagga 2275
                 ||||||||||||||||||||||||||||||||||||||||||||||||||||||||||||
Sbjct: 578604963 cgtaacgccgtccgccggtataaaaccggcctcccggcgccgagagacagaggaagagga 578605022

Query: 2276      acggacggcatagcgtagggaggggatccatccagatccaacagagcagaggagcgaccg 2335
                 ||||||||||||||||||||||||||||||||||||||||||||||||||||||||||||
Sbjct: 578605023 acggacggcatagcgtagggaggggatccatccagatccaacagagcagaggagcgaccg 578605082

Query: 2336      gtcggagcagagcagtggc 2354
                 |||||||||||||||||||
Sbjct: 578605083 gtcggagcagagcagtggc 578605101


Query= TdHDZipI-5B (promoter + 5’UTR sequence)(2039 letters)
Sequences producing significant alignments: chr2A (Sbjct)
Score = 2642 bits (1333), Expect = 0.0
Identities = 1369/1377 (99%), Gaps = 2/1377 (0%)
Strand = Plus / Plus

Query: 663       atcgacactaaccggcagtggcagtcgtagcccggtcttggcgtgctatcaacttttgtg 722
                 ||||||||||||||||||||||||||||||||||||||||||||||||||||||||||||
Sbjct: 637983129 atcgacactaaccggcagtggcagtcgtagcccggtcttggcgtgctatcaacttttgtg 637983188

Query: 723       tcgtgctacactgcggctcgaaatgacccgacccgcgcccgccgttaccgaaaccggaaa 782
                 |||||||||||||||||||||||||||||||||||||||||||||||||||| ||||||
Sbjct: 637983189 tcgtgctacactgcggctcgaaatgacccgacccgcgcccgccgttaccgaagccggaag 637983248

Query: 783       gaaactccaccagcgcactactacgagcgttcaacaaataaaggcagttcagtcgcaccg 842
                   ||||||||||||||||||||||||||||||||||||||||||||||||||||||||||
Sbjct: 637983249 --aactccaccagcgcactactacgagcgttcaacaaataaaggcagttcagtcgcaccg 637983306

Query: 843       ggtcaccgcttttaacttgcctgatctgctctagcacgtttggatcgtgggaggtcgggg 902
                 ||||||||||||||||||||||||||||||||||||||||||||||||||||||||||||
Sbjct: 637983307 ggtcaccgcttttaacttgcctgatctgctctagcacgtttggatcgtgggaggtcgggg 637983366

Query: 903       gcagcgaatcgccggcctccgggtggagaacgatcggtcggggataacctctgctccccg 962
                 ||||||||||||||||||||||||||||||||||||||||||||||||||||||||||||
Sbjct: 637983367 gcagcgaatcgccggcctccgggtggagaacgatcggtcggggataacctctgctccccg 637983426

Query: 963       cactgaccccgcaagtgctcgcctatcacgttcgcttcgcttcaccgtatctgtttatct 1022
                 ||||||||||||||||||||||||||||||||||||||||||||||||||||||||||||
Sbjct: 637983427 cactgaccccgcaagtgctcgcctatcacgttcgcttcgcttcaccgtatctgtttatct 637983486

Query: 1023      tgagttaccatgatgtcccttcctctttctctctcttttttctttctttttttgacatgg 1082
                 |||||||||||||||||||||||||| |||||||||||||||||||||||||||||||||
Sbjct: 637983487 tgagttaccatgatgtcccttcctctctctctctcttttttctttctttttttgacatgg 637983546

Query: 1083      tgatgtcccttcctggtaaacgatcaatcgagtggtaaactgggagtagtagtagaacgc 1142
                 ||||||||||||||||||||||||||||||||||||||||||||||||||||||||||||
Sbjct: 637983547 tgatgtcccttcctggtaaacgatcaatcgagtggtaaactgggagtagtagtagaacgc 637983606

Query: 1143      acagaggcgccaggcgacaacaggcgacaagacccgccaacgcgctagctgagtggcgtg 1202
                 ||||||||||||||||||||||||||||||||||||||||||||||||||||||||||||
Sbjct: 637983607 acagaggcgccaggcgacaacaggcgacaagacccgccaacgcgctagctgagtggcgtg 637983666

Query: 1203      ctgcgtgcatcggcccggcgaacgggacaaacgcggcgcgccacctcgacgccccttccg 1262
                 ||||||||||||||||||||||||||||||||||||||||||||||||||||||||||||
Sbjct: 637983667 ctgcgtgcatcggcccggcgaacgggacaaacgcggcgcgccacctcgacgccccttccg 637983726

Query: 1263      gcggaagcccccgcacacctggcgccgcgacggcgacgcgtgcagcccccgacggagcg 1322
                 ||||||||||||||||||||||||||||||||||||||||||||||||||||||||||||
Sbjct: 637983727 gcggaagcccccgcacacctggcgccgcgacggcgacgcgtgcagcccccgacggagcg 637983786

Query: 1323      cgcacggccattggtcctacctgttcctcctcccgcgcccgtccccgtctccccgtggaa 1382
                 ||||||||||||||||||||||||||||||||||||||||||||||||||||||||||||
Sbjct: 637983787 cgcacggccattggtcctacctgttcctcctcccgcgcccgtccccgtctccccgtggaa 637983846

Query: 1383      caccaaggcgcacacctttccaccacccattggccggcaagcccgccccatgatacacca 1442
                 ||||||||||||||||||||||||||||||||||||||||||||||||||||||||||||
Sbjct: 637983847 caccaaggcgcacacctttccaccacccattggccggcaagcccgccccatgatacacca 637983906

Query: 1443      ccggagtagtactacgtagtgctgatcctttcccgtgcagccgatcgatcgattcgtagt 1502
                 ||||||||||||||||||||||||||||||||||||||||||||||||||||||||||||
Sbjct: 637983907 ccggagtagtactacgtagtgctgatcctttcccgtgcagccgatcgatcgattcgtagt 637983966
```

```
Query: 1503      agatgaaaatgcaagaggttccgtggcgtggacagacagacataggtagagagaaataaa 1562
                 |||||||||||||||||||||||||||||||||||||||||||||| |||||||||||||
Sbjct: 637983967 agatgaaaatgcaagaggttccgtggcgtggacagacagacataggcagagagaaataaa 637984026

Query: 1563      cgaacgaaaattactcgtccggtagaggcttgcaattcgtaggccggaaggaagccttga 1622
                 |||| |||||||||||||||||||||||||||||||||||||||||||||||||||||||
Sbjct: 637984027 cgaatgaaaattactcgtccggtagaggcttgcaattcgtaggccggaaggaagccttga 637984086

Query: 1623      gtgagcggaaggaggaggaggagaagcaagatctttgcacccgtggccacgtttcgggtg 1682
                 |||||||||||||||||||| |||||||||||||||||||||||||||||||||||||||
Sbjct: 637984087 gtgagcggaaggaggaggagtagaagcaagatctttgcacccgtggccacgtttcgggtg 637984146

Query: 1683      ccgatctcgaggcgggaggatagacatgggggcgccgtggtgggcgcagcctctggcgcc 1742
                 ||||||||||||||||||||||||||||||||||||||||||||||||||||||||||||
Sbjct: 637984147 ccgatctcgaggcgggaggatagacatgggggcgccgtggtgggcgcagcctctggcgcc 637984206

Query: 1743      gctggatccgcgacacttggcccggggcgcgaaagcggctttcgcgcggtgtcaggccgc 1802
                 ||||||||||||||||||||||||||||||||||||||||||||||||||||||||||||
Sbjct: 637984207 gctggatccgcgacacttggcccggggcgcgaaagcggctttcgcgcggtgtcaggccgc 637984266

Query: 1803      gtgggcccggacggggaggtttctttatccgccgtcgcgaagaccacatgttcgcgtctc 1862
                 ||||||||||||||||||||||||||||||||||||||||||||||||||||||||||||
Sbjct: 637984267 gtgggcccggacggggaggtttctttatccgccgtcgcgaagaccacatgttcgcgtctc 637984326

Query: 1863      gcgcagcgcggacacgtaacgccgcccgccggtataaaacgggcctcccggcgccgagag 1922
                 ||||||||||||||||||||||||||||||||||||||||||||||||||||||||||||
Sbjct: 637984327 gcgcagcgcggacacgtaacgccgcccgccggtataaaacgggcctcccggcgccgagag 637984386

Query: 1923      acagaggaaggaggaagggacggacggcatagcgtagggaggggatccatccagcagagc 1982
                 ||||||||||||||||||||||||||||||||||||||||||||||||||||||||||||
Sbjct: 637984387 acagaggaaggaggaagggacggacggcatagcgtagggaggggatccatccagcagagc 637984446

Query: 1983      agaggagagcagcgaccggtcggagcagagcagtggcggcccactgggtcggtcggc 2039
                 |||||||||||||||||||||||||||||||||||||||||||||||||||||||||
Sbjct: 637984447 agaggagagcagcgaccggtcggagcagagcagtggcggcccactgggtcggtcggc 637984503
```

Fig. S3. Alignments of *TdHDZipI-5A* and *TdHDZipI-5B* promoter sequences and sequences of corresponding genes of *Triticum aestivum* cv. Chinese Spring, identified in the Whole Genome Reference Assembly Pseudomolecules v1.0 databases of the International Wheat Genome Sequencing Consortium, using the BLAST software (Altschul *et al*., 1997).

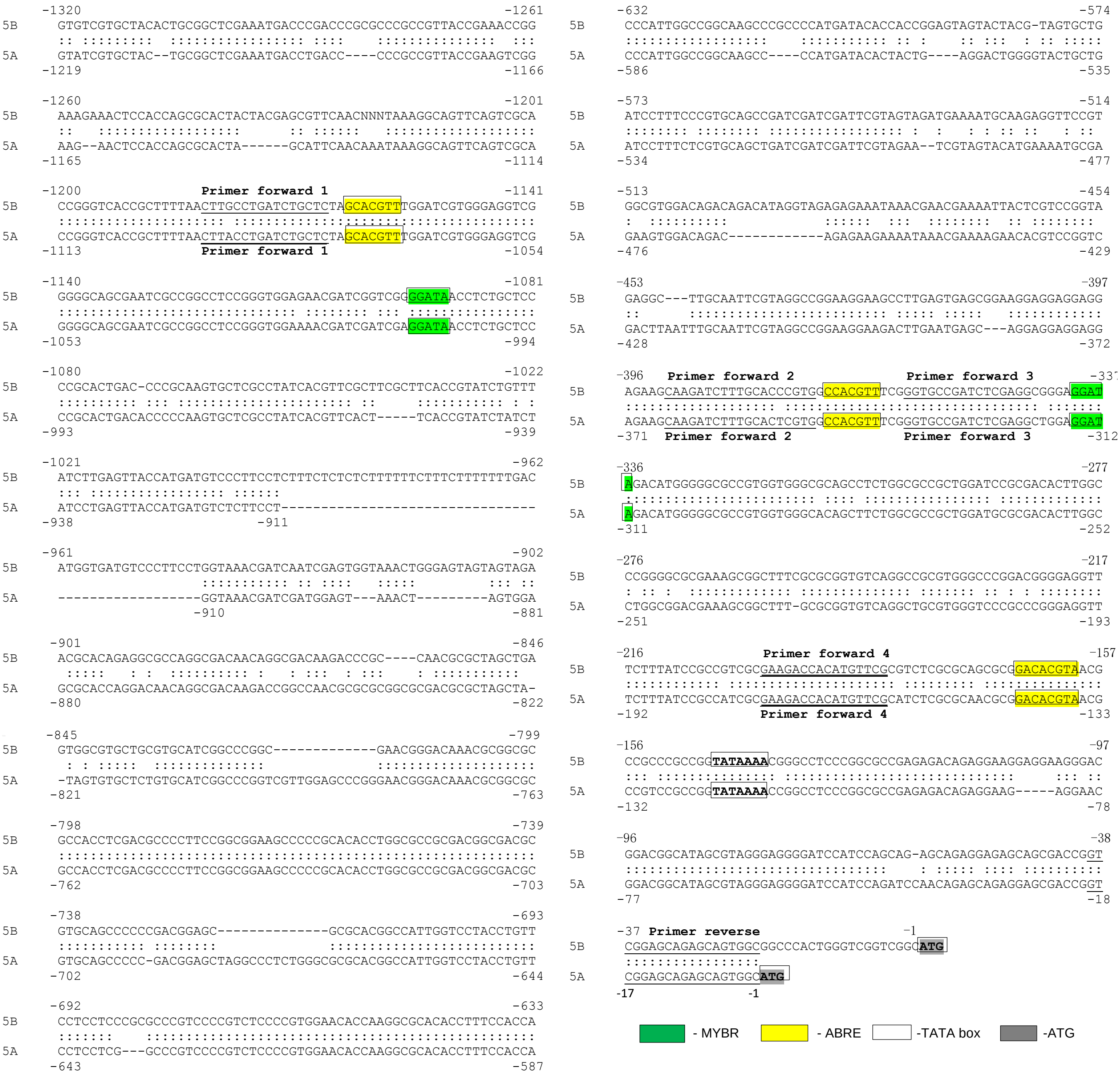

Fig. S4. Alignment of *TdHDZipI-5B* (5B) and *TdHDZipI-5A* (5A) promoters. LALIGN (Huang and Miller, 1991) was used to find the best local alignments. Primers used for generation of promoter deletions are underlined. Characteristic elements present in sequences of both promoters are indicated with boxes of different colours. MYBR - MYB recognition element; ABRE - abscisic acid responsive element; ATG - translational start.

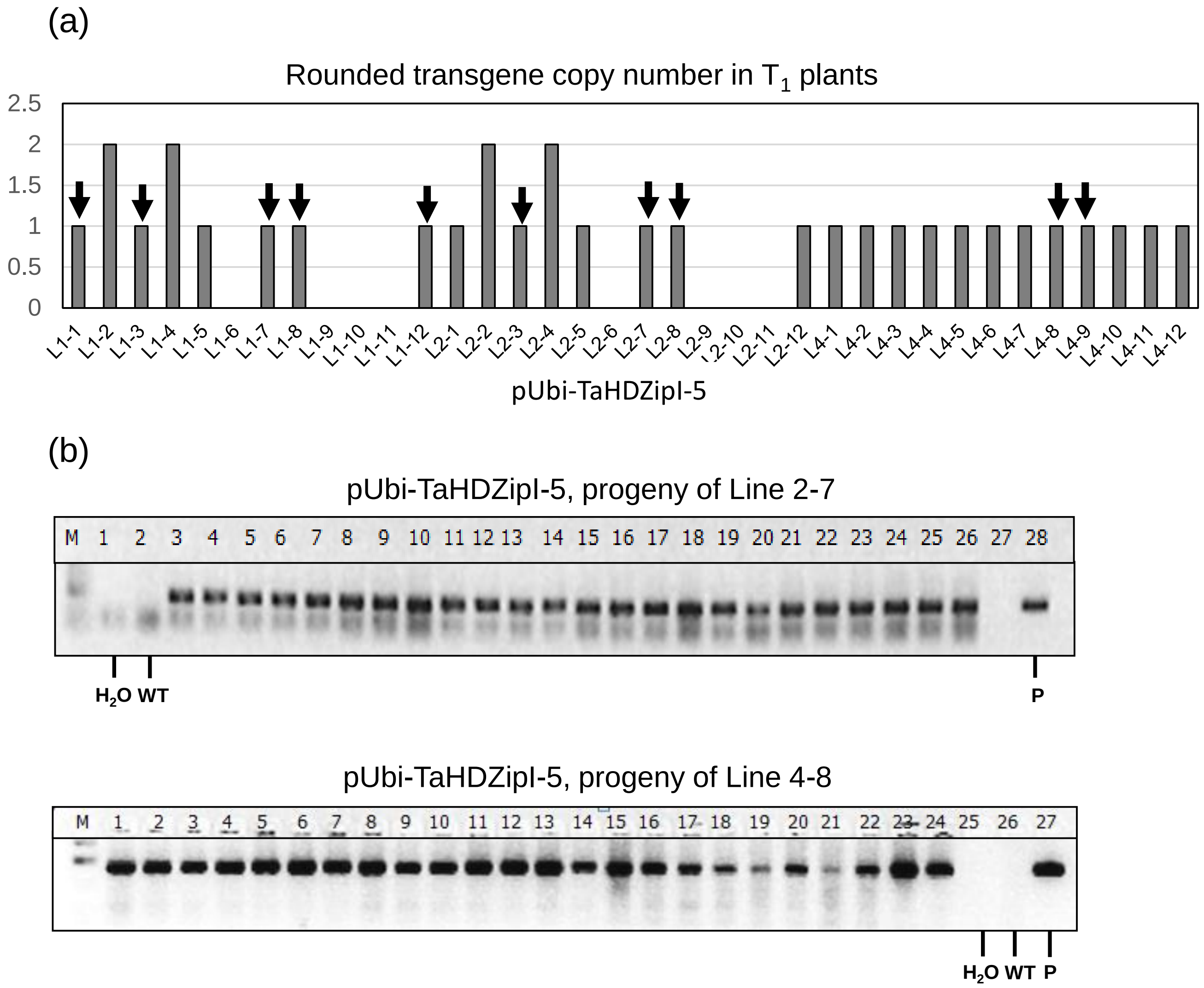

Fig. S5. (a) Transgene copy numbers in T_1 transgenic plants estimated by Q-PCR. Plants seeds used in analyses are indicated by arrows. (b) Examples of selection of homozygous lines by PCR using transgene-specific primers. Homozygous T_1 sublines of two independent single-transformation-event lines were selected using the analysis of the transgene (*TaHDZipI-5*) presence in the T_2 progeny. H_2O - sample containing no DNA, WT - sample containing DNA isolated from WT untransformed plant, P - positive control, where 1000-fold diluted plasmid DNA was used as a template.

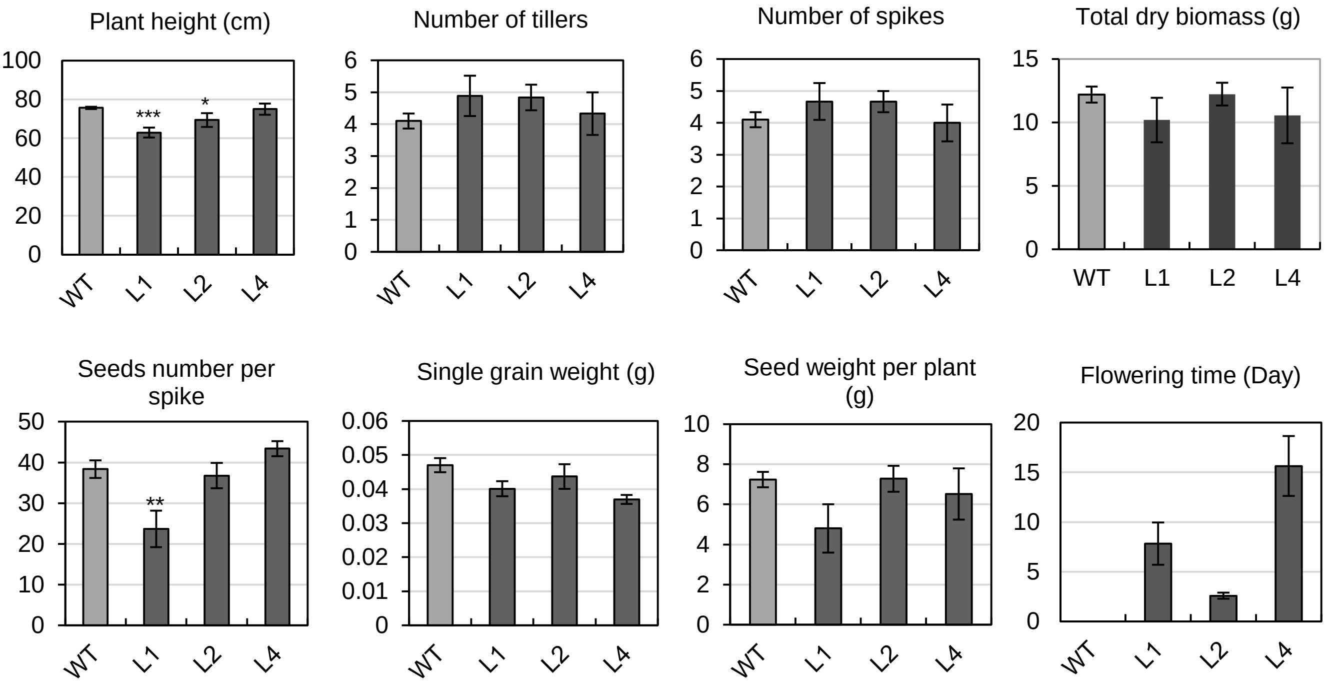

Fig. S6. Growth characteristics and yield components of control wild-type (WT) and transgenic T_1 wheat (*Triticum aestivum* cv. Gladius) plants transformed with pUbi-TaHDZipI-5. Plants were grown under well-watered conditions. Flowering time of transgenic plants was compared with the average flowering time of 12 control WT plants, which is represented as day 0. Differences between transgenic lines and WT plants were tested in the unpaired Student's t-test (* P<0.05, ** P<0.01, *** for P < 0.001).

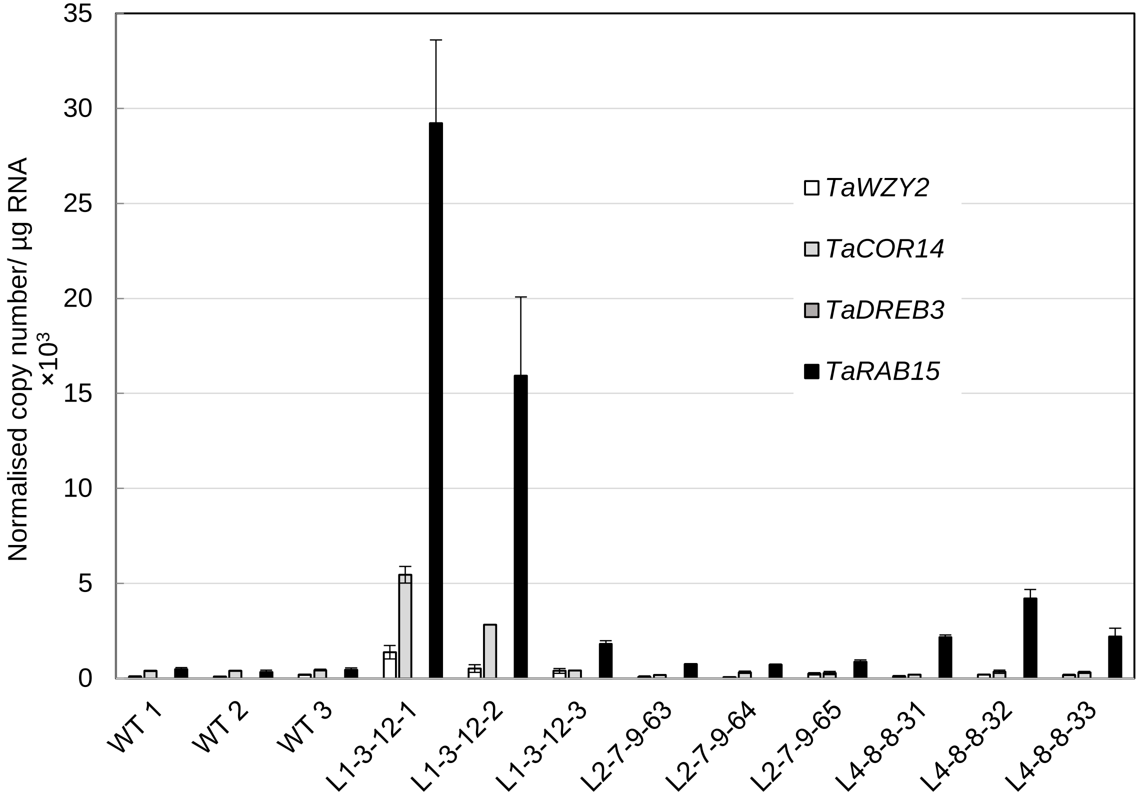

Fig. S7. Expression levels of three stress-inducible LEA (Late Embryogenesis Abundant)/COR (Cold-Responsive)/DHN (Dehydrin) genes (*TaWZY2*, GenBank: EU395844; *TaCOR14B*, GenBank: AF207546; *TaRab15*, GenBank: X59133) and the TaDREB3 (GenBank: DQ353853) regulatory gene, in leaves of unstressed control WT plants and T_3 sublines of tree independent transgenic lines. Expression levels of the *TaHDZipI-5* transgene in the same lines are shown in Fig. 6D. No correlation was found between the expression levels of the *TaHDZipI-5* transgene and downstream genes. Error bars represent ± SD of three technical replicates.

Table S1. List of PCR primers and DNA probes used in this study.

Short name	Purpose	Designation	Nucleotide sequence (5′–3′)
TaHDZipI-5, transgene	PCR, transgene specific primers	Forward primer	GGTCTTCCCGGAGTCCTTCTGC
		Reverse primer	CATCGCAAGACCGGCAACAGG
Nos terminator	Q-PCR, copy number	Forward primer	CTTAAGATTGAATCCT
		Reverse primer	CGAATTCAGTAACATAGA
		TaqMan probe	AGCGCGCAAACTAGGAT
Pin-b	Q-PCR, copy number	Forward primer	ATTTTCCAGTCACCTGGCCC
		Reverse primer	TGCTATCTGGCTCAGCTGC
		TaqMan probe	ATGGTGGAAGGGCGGCTGTGA
TaCRT	RT-qPCR	Forward primer	CGATGATGATGATGGCATATGG
		Reverse primer	TACCTGCCAAACTTCAATTCC
TaHDZipI-5, endogene	Q-PCR, gene expression	Forward primer	GAGAGCTGCGTGCTC
		Reverse primer	CGAACCACTTTGTACAAG
TaActin	Q-PCR, gene expression	Forward primer	GACAATGGAACCGGAATGGTC
		Reverse primer	GTGTGATGCCAGATTTTCTCCAT
TaCyclophilin	Q-PCR, gene expression	Forward primer	CAAGCCGCTGCACTACAAGG
		Reverse primer	AGGGGACGGTGCAGATGAA
TaGAPdH	Q-PCR, gene expression	Forward primer	TTCAACATCATTCCAAGCAGCA
		Reverse primer	CGTAACCCAAAATGCCCTTG
TaEFA	Q-PCR, gene expression	Forward primer	CAGATTGGCAACGGCTACG
		Reverse primer	CGGACAGCAAAACGACCAAG
TaWZY2	Q-PCR, gene expression	Forward primer	TACGGACAGCAAGGTCATACG
		Reverse primer	TTCTGCAGAGGTGTCCCAGAC
TaCOR14	Q-PCR, gene expression	Forward primer	TTCTGCAGAGGTGTCCCAGAC
		Reverse primer	ACCAACGTTGTAGCTCTTATAC
TaDREB3	Q-PCR, gene expression	Forward primer	CTCGATTCGCTTGCTCCTCAG
		Reverse primer	TCCTGATGACAAGCTGTAGTGTGC
TaRab15	Q-PCR, gene expression	Forward primer	ACAGGGACTGGAGGAGCCTAC
		Reverse primer	TGGAACTGGAAGGCTTTGACC
TdHDZipI-5 D1	PCR, promoter deletion fragment amplification	Forward primer	CACCCTTGCCTGATCTGCTC
		Reverse primer	GCCACTGCTCTGCTCCGAC
TdHDZipI-5 D2	PCR, promoter deletion fragment amplification	Forward primer	CACCCAAGATCTTTGCACCCGTG
		Reverse primer	GCCACTGCTCTGCTCCGAC
TdHDZipI-5 D3	PCR, promoter deletion fragment amplification	Forward primer	CACCGGTGCCGATCTCGAGG
		Reverse primer	GCCACTGCTCTGCTCCGAC
TdHDZipI-5 D4	PCR, promoter deletion fragment amplification	Forward primer	CACCGAAGACCACATGTTCG
		Reverse primer	GCCACTGCTCTGCTCCGAC

Table S2. A sequence alignment of 14 entries (with GenBank accessions) used to generate a phylogenetic tree displaying the evolutionary relationships of HD-Zip I γ-clade TFs from *Arabidopsis* and selected monocots, shown in Fig. 1. An asterisk (*) indicates positions with a single conserved residue; a colon (:) indicates conservation between groups of strongly similar properties (scoring > 0.5 in the Gonnet PAM 250 matrix); a full stop (.) indicates conservation between groups of weakly similar properties (scoring ≤ 0.5 in the Gonnet PAM 250 matrix).

```
AtHB7_AY091364           ----------------MTEGGEY-----------------------------SPAMMSAE
AtHB12_AY087187          ----------------MEEGDFF-----------------------------NCCFSEIS
Zmhdz4_KJ728250          ----------------MDRPDH----QQQQFFMPTTVQV-----PQPQQQQ-QQLCVPM-
Oshox22_AY224440         HASDGRTQLASWARIAMDRGDHHHLQQQHQFLMPPPAPV----VP-------PQLCMPAM
TaHDZipI-5_KT224376      ----------------MDY------HQQQQFLMPPPASL-----P----AQ-QQLCAPMM
Zmhdz6_EU972354          ----------------MERG-------DCQFTVVPPRQY----------DE-AQFMHQLM
Sb04g033380_XP_002452838 ----------------MERGD------DCQFMVV--HQY----------DEAAQLMHQLM
Oshox24_AK063685         ----------------MES--------DCQFLVAPPQPHMYYDTAAAAVDE-AQFLRQMV
TaHDZipI-4_KT224375      ----------------MES--------DRQFLLAPPPPM-----HAAPGDD-GQFLQQQQ
Zmhdz9_BT087258          ----------------MEG-D----------------------------DDGPEWMMEV-
Sb02g030660_XP_002462710 ----------------MDG-E----------------------------DDVPEWMMEV-
Zmhdz12_NM_001156038     ----------------MDGAE----------------------------DDGTEWMMH--
Oshox6_AK103160          ----------------MDG-E----------------------------ED-SEWMMMDV
TaHDZipI-3_KT224374      ----------------MEQGE----------------------------ED-GDWMMEP-
                                         *

AtHB7_AY091364           PFLTM-------------------KK--MKKSNHN--KNNQRRFSDEQIKSLEMMF-ESE
AtHB12_AY087187          SGMTMN-----------------KKK--MKKS------NNQKRFSEEQIKSLELIF-ESE
Zmhdz4_KJ728250          ----------LDEPPSSF-----LAG--RGGGGASGRGERKRRFTEEQIRSLESMFHAHH
Oshox22_AY224440         --MADEQYMDL--------GGGGAAA--APGRGGA--GERKRRFTEEQIRSLESMFHAHH
TaHDZipI-5_KT224376      -GMGMEMGMEMEMEEQLC-----FVG--RGGGGRG--AERKRRFTEEQTRSLESMFHAHH
Zmhdz6_EU972354          --AAGDQ-----QDP--A-----GAG--RGAAGGG--GERKRRFTEEQVRSLETTFHARR
Sb04g033380_XP_002452838 VAAAGDQ-----QDPNAG-----AAG--RGAGGGG--GERKRRFTEEQVRSLETTFHARR
Oshox24_AK063685         --AAADH-----HAAAAGRGGGDGDG--GGGGGGG--GERKRRFTEEQVRSLETTFHARR
TaHDZipI-4_KT224375      --LS---------------------------GGGA--GERKRRFTEEQVRSLESTFHTRR
Zmhdz9_BT087258          -------------------GGAGATG--KGKGGAL--DKNKKRFSEEQIKSLESMF-ATQ
Sb02g030660_XP_002462710 -------------------GGAGGKGGKGGGGGAL--DKNKKRFSEEQIKSLESMF-ATQ
Zmhdz12_NM_001156038     --------------------GAGGKG---KGGGAL--DKNKKRFSDEQTKSLESMF-ATQ
Oshox6_AK103160          -------------------GGKGGKG---GGGGGA--ADRKKRFSEEQIKSLESMF-ATQ
TaHDZipI-3_KT224374      -----------------------AAG---KKGGAM--IDRKKRFSEEQIKSLESMF-ATQ
                                                               ..::**::** :***  *   .

AtHB7_AY091364           TRLEPRKKVQLARELGLQPRQVAIWFQNKRARWKSKQLETEYNILRQNYDNLASQFESLK
AtHB12_AY087187          TRLEPRKKVQVARELGLQPRQVAIWFQNKRARWKTKQLEKEYNTLRANYNNLASQFEIMK
Zmhdz4_KJ728250          AKLEPREKAELARELGLQPRQVAIWFQNKRARWRSKQLEHDYALLRAKFDDLHAHVESLK
Oshox22_AY224440         AKLEPREKAELARELGLQPRQVAIWFQNKRARWRSKQLEHDYAALRSKYDALHSRVESLK
TaHDZipI-5_KT224376      AKLEPREKAELARELGLQPRQVAIWFQNKRARWRSKQLEHDFTALRADYDALHSRVESLK
Zmhdz6_EU972354          AKLEPREKAELARELGLQPRQVAIWFQNKRARWRSKQLEHDYAALRAQFDAMHARVESLR
Sb04g033380_XP_002452838 AKLEPREKAELARELGLQPRQVAIWFQNKRARWRSKQLEHDYAALRARYDALHARVDSLR
Oshox24_AK063685         AKLEPREKAELARELGLQPRQVAIWFQNKRARWRSKQIEHDYAALRAQYDALHARVESLR
TaHDZipI-4_KT224375      AKLEPREKAELARELGLQPRQVAIWFQNKRARWRSKQLEQDFAELRGHYDALRARVESLK
Zmhdz9_BT087258          TKLEPRQKLQLARELGLQPRQVAIWFQNKRARWKSKQLERDYSALRDDYDALLCSYESLK
Sb02g030660_XP_002462710 TKLEPRQKLQLARELGLQPRQVAIWFQNKRARWKSKQLEREYSALRDDYDALLCSYESLK
Zmhdz12_NM_001156038     AKLEPRQKLQLARELGLQPRQVAIWFQNKRARWKSKQLEREYSALRDDYHALLCSYESLK
Oshox6_AK103160          TKLEPRQKLQLARELGLQPRQVAIWFQNKRARWKSKQLEREYSALRDDYDALLCSYESLK
TaHDZipI-3_KT224374      TKLEPRQKLQLARELGLQPRQVAIWFQNKRARWKSKQLERQYAALRDDYDALLSSYDQLK
                         ::****:* ::**********************::**:* ::  **  :. : .  : ::

AtHB7_AY091364           KEKQALVSELQRLKEAT---QKKTQEEER-----QCSGD--------------------Q
AtHB12_AY087187          KEKQSLVSELQRLNEEM---QRPKEEKHH-----ECCGD--------------------Q
Zmhdz4_KJ728250          QDKLALTTQLSELSERL---RE-RDDRA--------------AAAGGGR----------E
Oshox22_AY224440         QEKLALTVQLHELRERL---RE-REER----------------SGNGGAA---------T
TaHDZipI-5_KT224376      HEKLALAAQLQELSERL---RE-RDG------------------GGGGAA---------T
Zmhdz6_EU972354          QEKIALAAQVDELRGRL---NE-RQDQ---------------------------------
Sb04g033380_XP_002452838 EEKLALAKQVDELRGRLQSVSE-RQDQ---------------------------------
Oshox24_AK063685         QEKLALADQVDELRGKL---NE-RQDQ---------------------------------
TaHDZipI-4_KT224375      QEKLTLAAQLEELKKKL---NE-RQDQ---------------------------------
Zmhdz9_BT087258          KEKHTLLKQLEKLAEML---HE-PRGKYSGNADAAGAGDDVRS-GVGGMK--DEFAD--A
```

```
Sb02g030660_XP_002462710 KEKHALLKQLEKLAEML---HE-PRGKYGGNAD-AGAGDDVRS-GVGGMK--EEFTDA-A
Zmhdz12_NM_001156038     DEKRALLKQLEKLAEML---HEPPQGKYYGG-----NADDVRSGGVGGTKEEEESTDACA
Oshox6_AK103160          KEKLALIKQLEKLAEML---QE-PRGKYGDN-----AGDDARSGGVAGMK-KEEFVGAGG
TaHDZipI-3_KT224374      KDKQALLNQLEKLAEML---RE-PGGAKCGDNAGAAARDDVRL-AVAGMSMKDEFVDAGG
                         .:* :*  :: .*          .

AtHB7_AY091364           AVVALSSTHHESENEENRRRKPEEVRPEMEMKDDKGH-HGVMCDHHDYE------DDDNG
AtHB12_AY087187          GLALSSST--ESHNG----------KSEPEGRLDQG---SVLC-------------NDGD
Zmhdz4_KJ728250          TMASSSSC--IGGGGEEEA------------EDDKRN--VLLFGCVDMEPPAESCVL-VG
Oshox22_AY224440         TAASSSSC--NGSGSEEVDD-----------DDDKRN---AAAGCLDLEPP-ESCVL-GG
TaHDZipI-5_KT224376      ATASSSSC--NGGGREL--------------DDDKRN-------VVDVEPP-ESCVL-GG
Zmhdz6_EU972354          ----SGSC--EVNDAEAA-------------ADDKRN-NSTSS-----------LVQ-DD
Sb04g033380_XP_002452838 ----SGSC--EVNDAA---------------DDGKRNLNSTTTTCLV-------LVQ-ED
Oshox24_AK063685         ----SGSC--DGGGAEGD-------------DDDKRN--SVMNASSS------GLVE-ED
TaHDZipI-4_KT224375      ----SASC--D---AAAE-------------VDDKSN-NVSSC-----------IVA-KD
Zmhdz9_BT087258          GAAPYSSE--GGGGG--------KF-AHF-TDDDV---GALFRPSS-----PQPSA--AG
Sb02g030660_XP_002462710 GAALYSSE--GGGGG--------KF-AHF-TDDDV---GALFRPSA------QPTA--AG
Zmhdz12_NM_001156038     GAALYSSE--CAGGG--------RFIAHFLADDDVGA-ALFRPPSS-----PQPTA--GL
Oshox6_AK103160          AATLYSSA--EGGGTTTTTTA--KLMPHF-GSDDVDA-GLFLRPSSQHHPPPPHAG--AG
TaHDZipI-3_KT224374      ASKLYSAS--EGCGGSG------KL-SLF-GEDDDDA-GLFLRPSL-----QLPTAHDGG
                              .:                          .

AtHB7_AY091364           YS--------NNIKREYFG-----GFEEEPD--H-LMNIVEPA-DSCLTSSDDWRGFKSD
AtHB12_AY087187          YN--------NNIKTEYF------GFEEETD--HELMNIVEKADDSCLTSSENWGGFNSD
Zmhdz4_KJ728250          ST-CAALA---DVSV-----------ESECDDQH-LH------------------YDDE
Oshox22_AY224440         AT-CATPA---DVSVE----------SDQCDD-Q-LD------------------YDEG
TaHDZipI-5_KT224376      TA-CGTPA---DVSAS---------VESECDD-H-LH------------------YDGA
Zmhdz6_EU972354          GA-TPPPA-AVDA--SEDSAAT----GEYYD--H-VA------------------YEYD
Sb04g033380_XP_002452838 DG-ATPPA-AVDASGSEDSAATEYGYGYDYD--HVVA------------------YGEG
Oshox24_AK063685         YV-SCLAVPVVDV--SEDGSAACGGSSYEYDH-H-LD------------------YLGG
TaHDZipI-4_KT224375      ES-AAPAA---DV---SDGSTP---GWYEYDN-H-LA------------------YGVD
Zmhdz9_BT087258          FT-SSGPP---EHQ--------------PFQF-H-SG---------C---------WPSS
Sb02g030660_XP_002462710 FTSSSGPP---EHQ--------------PFQF-H-SS--------SC---------WPSS
Zmhdz12_NM_001156038     LT-SSGPP---EHQ--------------PFQF-H-SG--------CC---------WPSS
Oshox6_AK103160          FT-SSEPA--ADHQ--------------SFNF-H-SS------------------WPSS
TaHDZipI-3_KT224374      FT-ASGPA---EYQQQ---------SPSSFPF-H-SN------------------WPSS
                                  :                      :                      :

AtHB7_AY091364           TTTLLDQSSNNYP-WRDF-WS----------
AtHB12_AY087187          --SLLDQSSSNYPNWWEF-WS----------
Zmhdz4_KJ728250          ---FPES-YCAMPELWEP-WPL--VEWNAVA
Oshox22_AY224440         L--FPES-FCATPELWEP-WPL--VEWNAVA
TaHDZipI-5_KT224376      V--FPES-FCATPELWEP-WPWPPVEWNAVA
Zmhdz6_EU972354          G--LHDPFVCATPDLWDT-WPL--LEWNAVA
Sb04g033380_XP_002452838 ---LHDP-LCATPDLWDT-WPL--LEWNAVA
Oshox24_AK063685         G-QLPDP-FCGMPDLWEI-WPM--VEWNAVA
TaHDZipI-4_KT224375      ---LQEP-FCATPELWETSWPL--VEWNAVA
Zmhdz9_BT087258          ----TEQ-TCSSSQWWEF-ESL--SE-----
Sb02g030660_XP_002462710 ---TTEQ-TCSSSQWWEF-ESL--SE-----
Zmhdz12_NM_001156038     ---SAEQ-TCSGSQWWEL-ESL--SE-----
Oshox6_AK103160          ----TEQ-TCSSTPWWEF-ESE---------
TaHDZipI-3_KT224374      ---AAEQ-TCSSSQWWEF-ESP--SE-----
                             :      .   :   .
```

Table S3. Hydrogen bonds in homo-dimeric TaHDZipI-3 and TaHDZipI-5, and hetero-dimeric TaHDZipI-3/TaHDZipI-5 with HDZ1 (5'-CAATCATTGC-3'/5'-GCAATGATTG-3').

Residues	Number of hydrogen bonds and distances in Å[1] HDZ1 (5'-CAATCATTGC-3'/5'-GCAATGATTG-3')								Water	DNA phospho-diester backbone	Total number
	A2	A3	T4	T7	T8	G11	T15	G16			
TaHDZipI-3											
Gln66	-	-	-	-	-	-	-	-	1 (3.0)	-	1
Asn73	-	-	-	-	2 (3.0, 3.4)	-	-	-	1 (2.8)	-	3
Lys79	-	-	-	-	-	-	-	-	-	1 (3.4)	1
TaHDZipI-3											
Gln66	-	-	-	-	-	-	-	-	2 (3.0, 2.8)	2 (3.4, 3.4)	4
Gln72	-	-	1 (3.2)	-	-	-	-	-	-	-	1
Asn73	1 (3.5)	-	-	-	-	-	-	-	-	-	1
Total	1	0	1	0	2	0	0	0	4	3	11

Residues	Number of hydrogen bonds and distances in Å[1] HDZ1 (5'-CAATCATTGC-3'/5'-GCAATGATTG-3')								Water	DNA phospho-diester backbone	Total number
	A2	A3	T4	T7	T8	G11	T15	G16			
TaHDZipI-5											
Arg58	-	-	-	1 (2.9)	-	-	-	1 (3.5)	-	-	2
Gln99	-	-	-	-	-	-	-	-	1 (3.0)	1 (2.6)	2
Gln105	-	-	-	-	1 (3.4)	-	-	-	1 (3.1)	-	2
Asn106	-	-	-	-	1 (3.4)	-	-	-	1 (3.2)	-	2
TaHDZipI-5											
Arg60	-	-	-	-	-	-	-	-	-	1 (2.3)	1
Gln99	-	-	-	-	-	-	-	-	1 (3.0)	1 (3.3)	2
Asn106	-	2 (3.0, 3.1)	-	-	-	-	-	-	1 (2.8)	-	3
Total	0	2	0	1	2	0	0	1	5	3	14

Residues	Number of hydrogen bonds and distances in Å[1] HDZ1 (5'-CAATCATTGC-3'/5'-GCAATGATTG-3')								Water	DNA phospho-diester backbone	Total number
	A2	A3	T4	T7	T8	G11	T15	G16			
TaHDZipI-3											
Arg26	-	-	-	1 (3.1)	-	-	1 (3.5)	-	-	-	2
Lys27	-	-	-	-	-	-	-	1 (3.0)	-	-	1
Arg65	-	-	-	-	-	-	-	-	-	1 (3.2)	1
Gln66	-	-	-	-	-	-	-	-	1 (3.0)	2 (2.6, 3.2)	3
Gln72	-	-	-	-	1 (3.4)	1 (2.9)	-	-	1 (2.8)	-	3
Asn73	-	-	-	-	1 (2.3)	-	-	-	1 (3.2)	-	2
Arg77	-	-	-	-	-	-	-	-	-	1 (3.2)	1
TaHDZipI-5											
Arg60	-	-	-	-	-	-	-	-	-	1 (2.8)	1
Gln99	-	-	-	-	-	-	-	-	2 (3.0, 3.2)	1 (3.4, 3.4)	3
Asn106	1 (2.9)	2 (3.2, 3.3)	-	-	-	-	-	-	1 (2.8)	-	4
Arg110	-	-	-	-	-	-	-	-	-	1 (3.1)	1
Total	1	2	0	1	2	1	1	1	6	7	22

Table S4. Characteristics of the T_2/T_3 progenies of *TaHDZipI-5* transgenic lines analysed in large containers under well-watered or mild drought condition.

Construct	Container No.	Condition	Lines	Total number of plants	Number of dead plants	Number of nulls	Number of plants with expressed transgene
**pUbi-TaHDZipI-5**	17	Drought	WT	16	0	-	-
			L1-3-9	16	3	0	13
			L2-7-9	16	0	0	16
			L4-8-9	16	0	0	16
	18	Well-watered	WT	16	0	-	-
			L1-3-9	16	3	0	13
			L2-7-9	16	0	0	16
			L4-8-9	16	0	0	16
**pWRKY71-TaHDZipI-5**	21	Drought	WT	16	0	-	-
			L14-7	16	0	4	12
			L46-3	16	0	3	13
			L48-7	16	0	5	11
	22	Well-watered	WT	16	0	-	-
			L14-7	16	0	2	14
			L46-3	16	2	1	13
			L48-7	16	0	8	8
**pCor39-TaHDZipI-5**	23	Drought	WT	16	0	-	-
			L23-4	16	1	4	11
			L39-7	16	0	0	16
			L55-4	16	0	6	10
	24	Well-watered	WT	16	0	-	-
			L23-4	16	4	0	12
			L39-7	16	0	0	16
			L55-4	16	0	2	14
